# VEGFD/VEGFR2 axis induces the dedifferentiation of high endothelial venules and impairs lymphocyte homing

**DOI:** 10.1172/jci.insight.191041

**Published:** 2025-07-22

**Authors:** Weichang Yang, Juan Wu, Shanshan Cai, Hongquan Xing, Jiajia Xiang, Xinyi Zhang, Xiaoyan Su, Xiaoqun Ye

**Affiliations:** 1 Department of Respiratory and Critical Care Medicine, The Second Affiliated Hospital of Nanchang University, JiangxiMedical College, Nanchang University, Nanchang, Jiangxi, China.; 2 Jiangxi Key Laboratory of Molecular Medicine, Nanchang, Jiangxi, China.; 3 Department of Pathology, The Second Affiliated Hospital of Nanchang University, Jiangxi, Medical College, Nanchang University, Nanchang, Jiangxi, China.

**Keywords:** Immunology, Pulmonology, Cancer, Lymph

## Abstract

High endothelial venules (HEVs) are important structures in lymph nodes (LNs) that mediate lymphocyte homing, and their dedifferentiation is a necessary step before LN metastasis. Whether vascular endothelial growth factor–related (VEGF-related) signaling, which plays an important role in LN metastasis, is involved in the dedifferentiation of HEVs remains unclear. Here, we confirmed increased expression of VEGFA, VEGFC, and VEGFD; HEV dedifferentiation; and impaired lymphocyte homing function in tumor-draining LNs (TDLNs). Furthermore, we demonstrated that tumor-secreted VEGFA induced lymphangiogenesis in TDLNs to promote premetastatic niche (PMN) formation; VEGFC promoted HEV proliferation but did not affect its lymphocyte homing function. Notably, we showed that VEGFD induced the dedifferentiation of HEVs by binding to VEGFR2 on the endothelial surface of HEVs and further impaired the lymphocyte homing function of TDLNs. Overall, we revealed that tumor-secreted VEGFD interacted with VEGFR2, induced HEV dedifferentiation, and reduced lymphocyte homing, providing potential insights for the prevention and treatment of LN metastasis.

## Abstract 2

In this study, we confirmed the mechanism by which VEGFD/VEGFR2 regulates high endothelial venule mediated decrease in lymphocyte homing function and promotes lymph node pre-metastatic niche.

## Graphical abstract

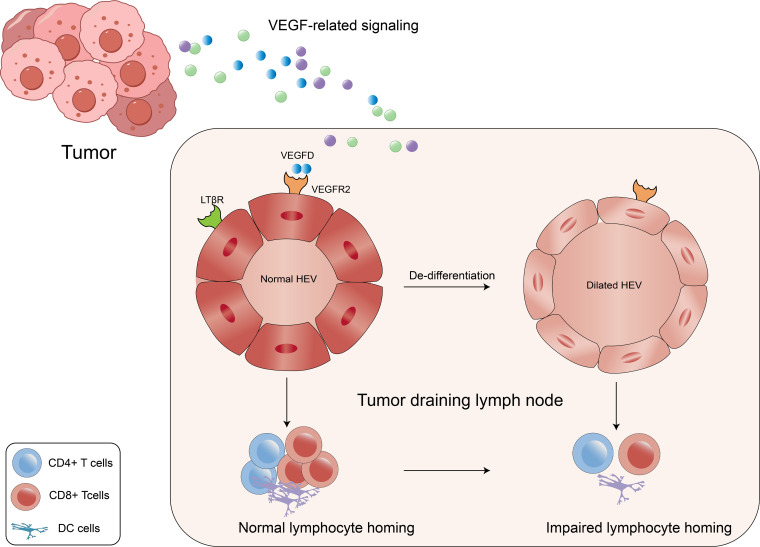

## Introduction

Lymph nodes (LNs) are the most common and earliest site of tumor metastasis, and exploring the mechanisms of LN metastasis contributes to the development of therapeutic strategies for tumors (1). In 2005, Lyden and colleagues first proposed that the premetastatic niche (PMN) based on the “seed and soil” hypothesis (2). They suggest that tumors induce a series of changes in metastatic organs (such as the LN) that are adapted to the growth of tumor cells in preparation for their arrival (3), which helps us to better understand the mechanisms of LN metastasis.

The vascular endothelial growth factor (VEGF) family, which comprises tumor-derived secreted factors (TDSFs), plays an important role in LN metastasis (4). VEGF-related signaling usually functions in conjunction with its corresponding receptors (VEGFR1, VEGFR2, and VEGFR3) (5). Previous studies report that VEGFA, VEGFC and VEGFD play roles in tumor metastasis by promoting tumor lymphangiogenesis or angiogenesis and further promoting LN metastasis (6–8). However, these studies lack direct evidence for the involvement of the VEGF family (VEGFA/C/D) in LN metastasis, and the specific mechanisms by which VEGF modulates the LN microenvironment and promotes PMN formation remain unclear.

The tumor-draining lymph node (TDLN) is the primary site where LN metastasis occurs, and its presence is strongly associated with both the prognosis and immune response of patients with tumors (9). Generally, the LN is an important immune organ; when a pathogen invades, the associated antigen can enter the LN with the lymphatic fluid through the lymphatic vessels to generate an immune response and destroy the pathogen (10). Recent studies have reported that the TDLN microenvironment undergoes a series of alterations before tumor cells metastasize and that it progressively loses its normal immune response function, which directly leads to the arrival of tumor cells (11). Lymphatic vessel dilatation, high endothelial vessel (HEV) dedifferentiation, and fibroblastic reticular cell (FRC) fibrosis and remodeling are thought to characterize the altered microenvironment of the TDLN (9); however, the intrinsic links and underlying mechanisms of these structural changes are unclear.

HEVs are highly specialized blood vessels composed of high endothelial cells (HECs), which present a plump, columnar morphology that distinguishes them from other vascular endothelial cells (12). A variety of monoclonal antibodies have been developed for the recognition of HEVs, of which MECA-79 is considered the best marker for the recognition of HEVs (13). In addition, HEVs are important channels that mediate lymphocyte homing and play important roles in maintaining LN microenvironmental homeostasis (14). Qian et al. observed HEV dedifferentiation (including luminal expansion and thinning of the tubular wall) in patients with breast cancer and concluded that this change was manipulated by tumor cells (15). However, a further explanation of HEV dedifferentiation is lacking.

Here, we obtained direct evidence for the involvement of VEGF-related signaling in HEV dedifferentiation and impaired lymphocyte homing function by constructing a TDLN model. We found that tumor-secreted VEGFD binds to VEGFR2 on the surface of HEVs, impairs the lymphocyte homing function of HEVs, reduces the immune response of TDLNs, and provides favorable conditions for the arrival of tumor cells.

## Results

### HEV expansion and decreased lymphocyte content in human metastatic LNs.

**Figure 1:**
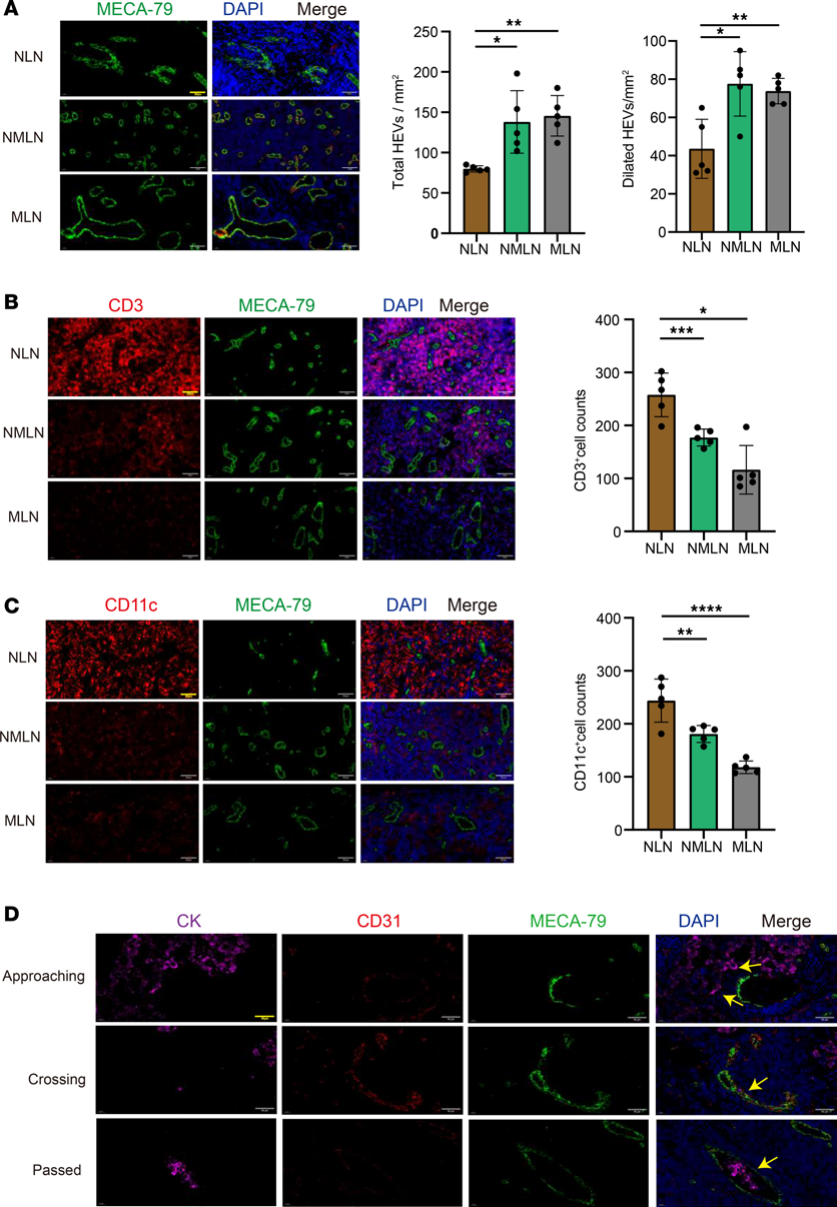
HEV expansion and decreased lymphocyte content in lung cancer lymph node. (**A**) Immunohistofluorescence (IF) staining of the total high endothelial venules (HEVs) and dilated HEVs (MECA-79, green) in normal lymph node (NLN), nonmetastatic lymph node (NMLN), and metastatic lymph node (MLN) (*n* = 5). (**B**) IF staining of CD3 (red) and MECA-79 (green) in NLN, NMLN, and MLN (*n* = 5). (**C**) IF staining of CD11c (red) and MECA-79 (green) in NLN, NMLN, and MLN (*n* = 5). (**D**) Lung cancer cells approaching, crossing, and passing the HEVs. In this figure, data are shown as mean ± SD. Yellow arrows, lung cancer cells. (**A**–**C**) *P* values were measured by 1-way ANOVA with Tukey’s multiple-comparison test. *P < 0.05, **P < 0.01, ***P < 0.001, ****P < 0.0001. Scale bars: 50 μm (**A**–**D**).

We collected 10 lymph node samples (5 from lung cancer patients and 5 from normal patients) to investigate the changes in the HEVs within the LNs, and the basic characteristics of the lung cancer patients are shown in Supplemental Table 1 (supplemental material available online with this article; https://doi.org/10.1172/jci.insight.191041DS1). The results show that the number of HEVs in the normal lymph nodes (NLNs) was 79.8 ± 3.8, which was substantially lower than that in the nonmetastatic lymph nodes (NMLNs) (138 ± 38.8) and metastatic lymph nodes (MLNs) (145.6 ± 25.2). The number of dilated HEVs was 43.6 ± 15.5 in NLNs and 77.6 ± 16.9 and 73.8 ± 6.7 in NMLNs and MLNs, respectively, and the proportion of dilated HEVs was higher in NMLNs and MLNs (Figure 1A), indicating substantial alterations in HEVs in patients with one or multiple tumors. Based on previous reports of HEV morphological characteristics (15), we confirmed that HEVs in NMLNs and MLNs underwent dedifferentiation. We examined the degree of HEV-mediated T cell and DC homing to assess the effects of HEV alterations on lymphocyte homing. We confirmed that the numbers of CD3^+^ T cells (Figure 1B) and DCs (Figure 1C) were substantially reduced in NMLNs and MLNs compared with NLNs. Previous studies suggest a gradual loss of HEV function before tumor LN metastasis, and we describe 3 states of lung cancer cells (cytokeratin [CK]) approaching, crossing, and passing through the HEVs in the MLNs (Figure 1D). Our findings reveal that tumor cells progressively approached HEVs, penetrated the vessel wall, and entered the lumen, facilitating their further dissemination. The above results suggest that changes in the morphology and function of HEVs in LNs from patients with lung cancer lead to impaired homing of LNs, but the associated mechanisms need to be further investigated.

### VEGF-related signaling molecule expression in the TDLNs.

We established a TDLN mouse model (Supplemental Figure 1A) by injecting Lewis lung carcinoma (LLC) cells into C57BL/6J mice and A549 cells into BALB/c-nu mice to explore the mechanisms involved in HEV loss and reduced lymphocyte numbers in TDLNs. We found no significant difference in the TDLN volume between the LLC+C57BL/6J and A549+BALB/c-nu groups (Supplemental Figure 1B). Additionally, on Day 21 after tumor cell inoculation (when the C57BL/6J mice had reached ethical endpoints), we assessed the TDLN metastasis rate in C57BL/6J and BALB/c-nu mice (Supplemental Figure 1C). The results show that the LN metastasis rate in the LLC+C57BL/6J group was higher than that in the A549+BALB/c-nu group (Supplemental Figure 1D). Therefore, subsequent studies were conducted using the LLC+C57BL/6J TDLN mouse model.

**Figure 2:**
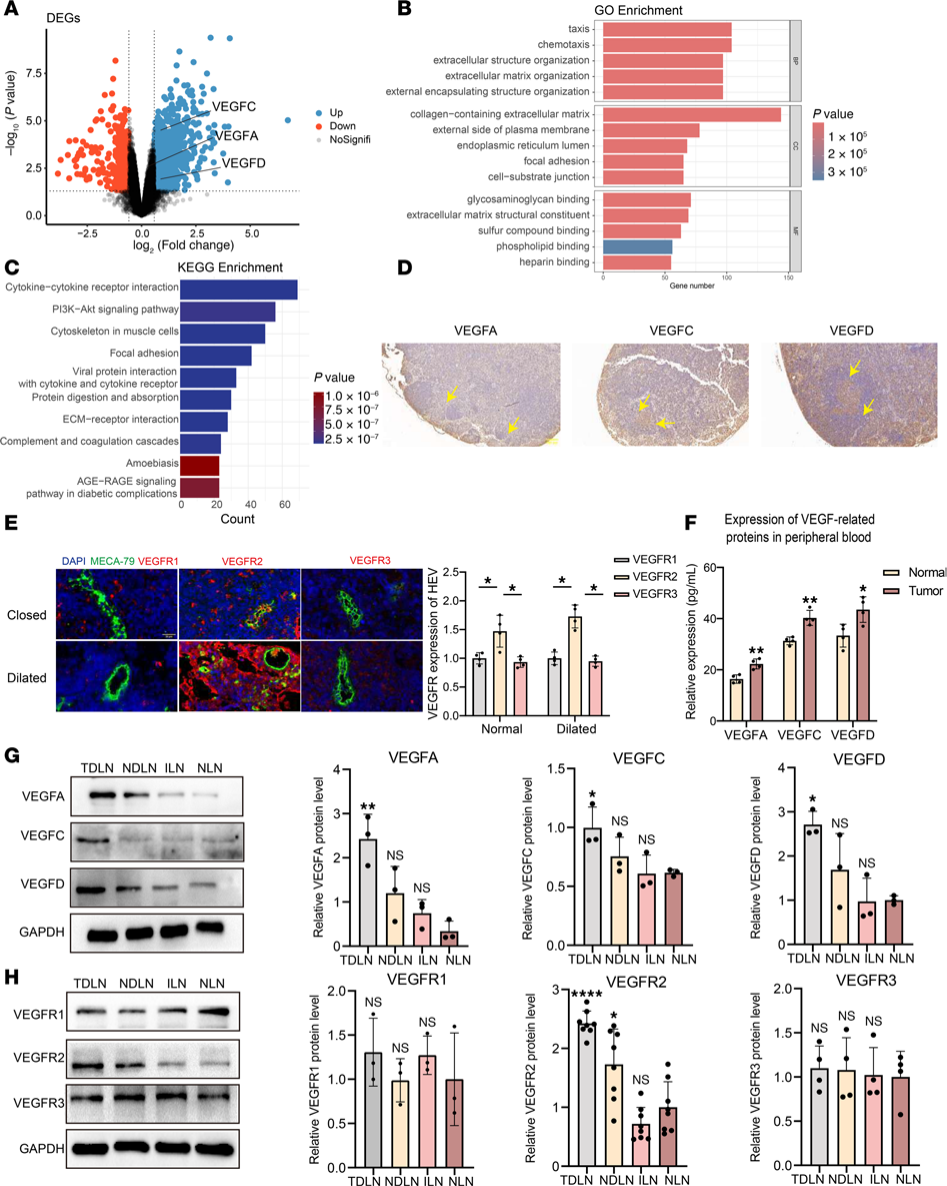
VEGF-related proteins expression in TDLN. (**A**) Differential gene expression analysis between tumor draining lymph node (TDLN) and normal lymph node (NLN). (**B**) GO enrichment analysis of differentially expressed genes (DEGs). (**C**) KEGG enrichment analysis of DEGs. (**D**) IHC staining of VEGFA, VEGFC and VEGFD in TDLN. (**E**) Immunohistofluorescence staining of VEGFR1 (green), VEGFR2 (green), VEGFR3 (green), and MECA-79 (green) in TDLN (*n* = 4). (**F**) ELISA for VEGFA, VEGFC, and VEGFD in peripheral blood between normal and tumor mouse (*n* = 4). (**G**) Western blotting (WB) assay of VEGFA, VEGFC, and VEGFD in TDLN, nondraining lymph node (NDLN), inguinal lymph node (ILN), and NLN (*n* = 3). (**H**) WB assay of VEGFR1 (*n* = 3), VEGFR2 (*n* = 8), and VEGFR3 (*n* = 4) expression in TDLN, NDLN, ILN, and NLN. Data are shown as mean ± SD (**E**–**H**). (**E**, **G**, and **H**) *P* values were measured by 1-way ANOVA with Tukey’s multiple-comparison test. (**F**) P values were measured by unpaired, 2-tailed Student’s *t* test with or without Welch’s correction analysis. *P < 0.05, **P < 0.01, ****P < 0.0001. Scale bar: 20 μm (**E**).

Next, we explored the regulatory mechanisms in TDLNs by analyzing the transcriptomic profiles of TDLNs and NLNs. The differential expression analysis confirmed that VEGFC and VEGFD were upregulated in TDLNs, whereas VEGFA expression was not substantially different (Figure 2A). The gene ontology (GO) enrichment analysis revealed the significant involvement of chemotaxis, taxis, and extracellular matrix organization in the biological process category. In the cellular component category, the collagen-containing extracellular matrix, the external side of the plasma membrane, and the endoplasmic reticulum lumen were substantially enriched. In the molecular function category, glycosaminoglycan binding, extracellular matrix structural constituents and sulfur compound binding were substantially enriched (Figure 2B). The Kyoto Encyclopedia of Genes and Genomes (KEGG) enrichment analysis revealed the significant enrichment of pathways related to cytokine-cytokine receptor interactions, the PI3K/Akt signaling pathway, and ECM-receptor interactions (Figure 2C). These results indicate a significant enrichment of angiogenesis-related functions and lymphocyte chemotaxis in TDLNs. We found that VEGFA, VEGFC, and VEGFD were widely expressed in TDLNs (Figure 2D), especially in the LN paracortical region. Similarly, we assessed VEGFR1, VEGFR2, and VEGFR3 expression on closed and dilated HEVs. We found that the expression of VEGFR2 was substantially higher than that of VEGFR1 and VEGFR3 (Figure 2E), suggesting that VEGF-related proteins are more likely to be involved in HEV regulation by binding to VEGFR2 rather than VEGFR1 or VEGFR3. We explored the expression of the VEGFA, VEGFC, and VEGFD proteins in lung cancer and adjacent tissues and showed that VEGFA, VEGFC, and VEGFD were substantially highly expressed in lung cancer tissues (Supplemental Figure 2A), which may be the main source of VEGF-related protein expression in TDLNs. Additionally, compared with normal mice, tumor-bearing mice presented substantially higher levels of VEGFA (22.3 versus 16.4 pg/mL), VEGFC (40.3 versus 31.3 pg/mL), and VEGFD (43.5 versus 33.4 pg/mL) in the peripheral blood (Figure 2F), indicating that these factors were secreted into the TDLN. The expression of VEGFA, VEGFC, and VEGFD was substantially higher in TDLNs than in NLNs, although significant differences were not observed between nondraining lymph nodes (NDLNs) and right inguinal lymph nodes (ILNs) (Figure 2G), suggesting that the expression of VEGF-related proteins was elevated in TDLNs before LN metastasis. VEGFR2 expression was also increased in TDLNs, whereas VEGFR3 expression was not substantially changed (Figure 2H). These results suggest that VEGF-related proteins and their receptors were secreted into the TDLN before lung cancer LN metastasis, making a further exploration of their relationships necessary.

### Impaired lymphocyte homing of HEVs in TDLNs.

**Figure 3:**
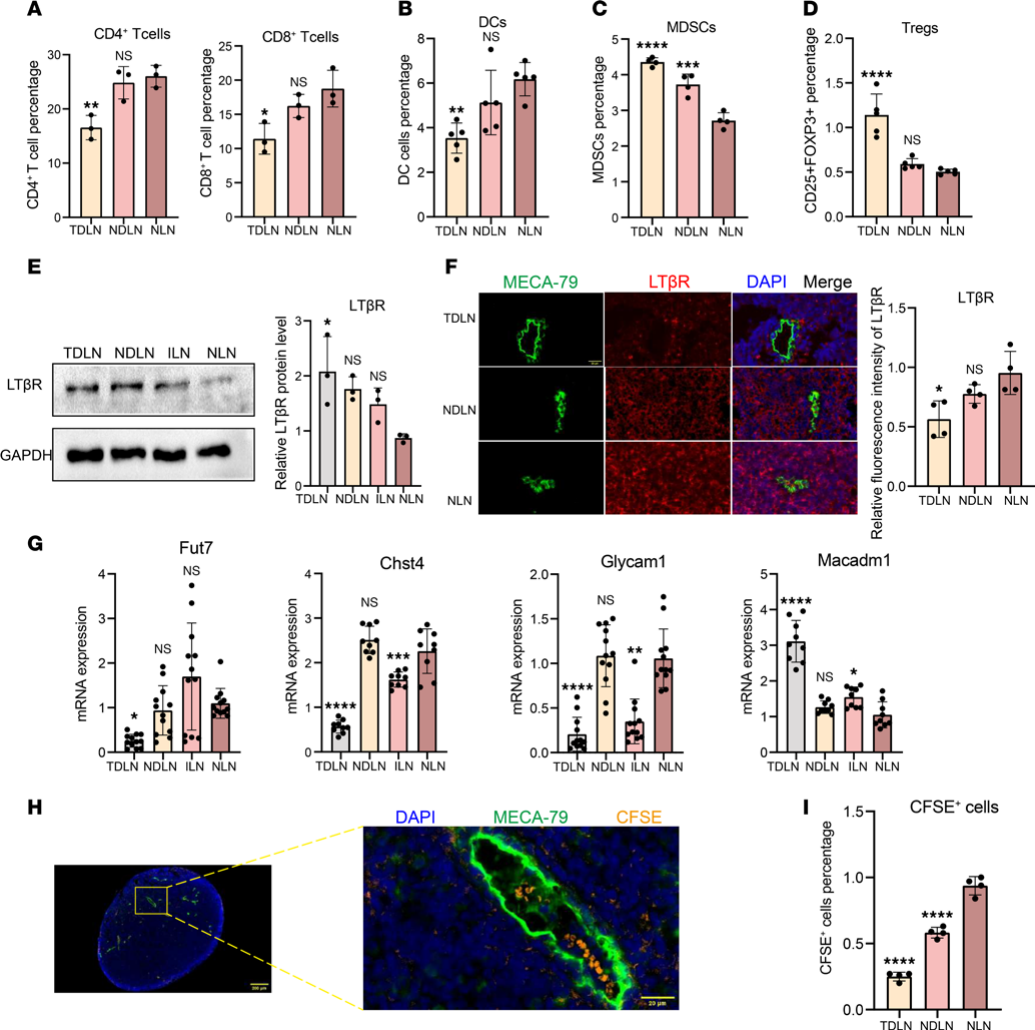
Impaired lymphocyte homing of HEV in TDLN. (**A**) Flow cytometry (FCM) of CD4^+^ and CD8^+^ T cell expression between tumor draining lymph node (TDLN), nondraining lymph node (NDLN), and mouse normal lymph node (NLN) (*n* = 3). (**B**) FCM of DCs in each group (*n* = 5). (**C**) FCM of myeloid-derived suppressor cells in each group (*n* = 4). (**D**) FCM of Tregs in each group (*n* = 5). (**E**) Western blotting (WB) assay of total LTβR expression between TDLN, NDLN, ILN and NLN (*n* = 3). (**F**) Immunohistofluorescence (IF) staining of LTβR (red) and MECA-79 (green) in each group (*n* = 4). (**G**) Quantitative reverse transcription polymerase chain reaction assays of the expression of Fut7 (*n* = 12), Chst4 (*n* = 9), Glycam1(*n* = 12), and Macadm1(*n* = 9) in the TDLN, NDLN, and NLN. (**H**) IF staining of CFSE (orange) and MECA-79 (green) in the TDLN. (**I**) FCM of CFSE^+^ cells in TDLN, NDLN, and NLN (*n* = 4). Data are shown as mean ± SD (**A**–**G** and **I**). (**A**–**G** and **I**) *P* values were measured by 1-way ANOVA with Tukey’s multiple-comparison test. *P < 0.05, **P < 0.01, ***P < 0.001, ****P < 0.0001. Scale bars: 20 μm (**F** and **H**) and 200 μm (**H**).

We compared lymphocyte populations in TDLNs, NDLNs, and NLNs to examine the link between reduced lymphocyte counts and HEVs in TDLNs. The percentages of CD4^+^ T cells were 17.24%, 24.83%, and 25.25%, respectively; the percentages of CD8^+^ T cells were 11.68%, 15.54%, and 18.33% (Figure 3A); and the percentages of DCs were 3.73%, 5.91%, and 5.71% (Figure 3B). In addition, we examined the numbers of immunosuppressive cells such as MDSCs and Tregs. The results showed that the proportion of MDSCs was higher in TDLNs (4.36%) and NDLNs (3.73%) than in NLNs (2.72%) (Figure 3C), and the proportion of Tregs was higher in TDLNs (1.40%) than in NLNs (0.59%), but a significant difference was not observed in NDLNs (0.50%) or NLNs (Figure 3D). The decrease in the number of immune cells and increase in the number of immunosuppressive cells indicated an impaired immune response in the TDLNs.

We analyzed changes in the TDLNs on Days 3, 10, and 18 after tumor formation. The HEV morphology shifted from tall columnar shapes to dilated lumens with thinner walls (Supplemental Figure 2B), whereas the numbers of CD4^+^ T cells and DCs progressively decreased (Supplemental Figure 2C). We further analyzed VEGF signaling and receptor dynamics and observed a progressive increase in VEGFA/C/D levels as the tumor progressed, with notable upregulation of VEGFR2 by Day 18 (Supplemental Figure 2D). These findings suggest that PMN formation in TDLNs is a gradual process closely linked to VEGF signaling, lymphocyte depletion, and HEV alterations.

Next, we analyzed lymphocyte homing changes in HEVs within TDLNs. LTβR is crucial for HEV maturation (16), and the results show that total LTβR protein expression was increased in TDLNs compared with NLNs but was not substantially different between NDLNs and ILNs (Figure 3E). Further studies reveal that LTβR expression around dilated HEVs was decreased (Figure 3F). These findings suggest that LTβR may play different roles on TDLN and HEV surfaces. We confirmed the downregulation of the HEV maturation markers Fut7, Glycam1, and Chst4 and the upregulation of the immature marker Macadm1 (Figure 3G), indicating an immature state of HEVs in TDLNs. Further analysis of lymphocyte homing revealed that 5,6-carboxyfluorescein diacetate succinimidyl ester (CFSE^+^) cells migrated through HEVs into the LN (Figure 3H), with TDLNs showing substantially fewer CFSE^+^ cells than NLNs and NDLNs (Figure 3I).

### VEGFA is not involved in HEV dedifferentiation or lymphocyte homing.

**Figure 4:**
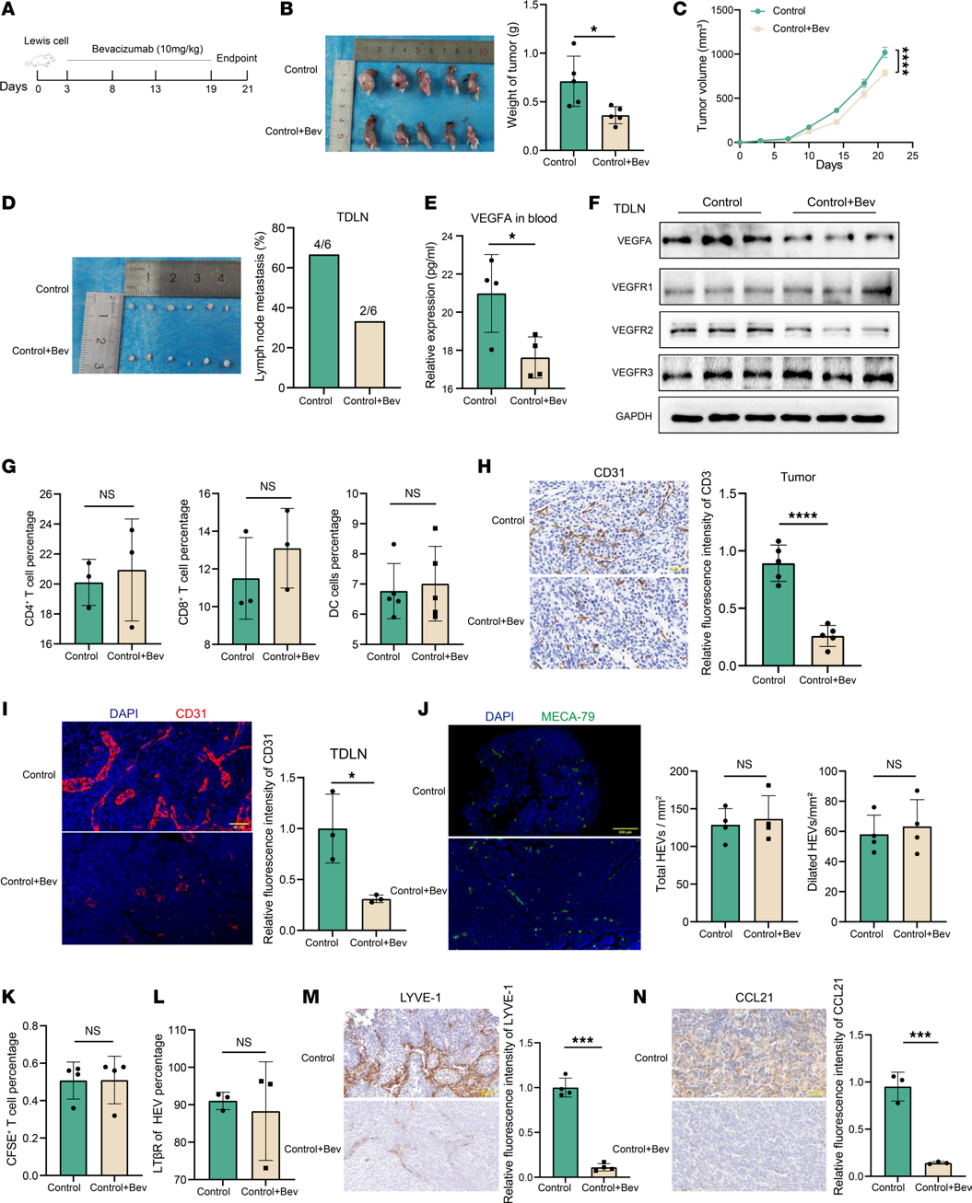
VEGFA was not involved in HEV dedifferentiation and lymphocyte homing. (**A**) Bevacizumab treatment protocol. (**B**) Tumor weight in the control and bevacizumab group (*n* = 5). (**C**) Tumor growth curves in the control and bevacizumab groups (*n* = 5). (**D**) Tumor draining lymph node (TDLN) metastasis rate in the control and bevacizumab groups (*n* = 6). (**E**) ELISA of VEGFA expression in peripheral blood between control and bevacizumab groups (*n* = 4). (**F**) Western blotting assay of VEGFA and its related receptor expression in TDLN between the bevacizumab and control groups (*n* = 3). (**G**) Flow cytometry (FCM) of the percentage of CD4^+^ (*n* = 3), CD8^+^ (*n* = 3) T cells, and DCs (*n* = 5) in TDLN between the bevacizumab and control group. (**H**) IHC staining of CD31 expression in tumor tissue between 2 groups (*n* = 5). (**I**) Immunohistofluorescence (IF) staining of CD31 (red) in tumor between 2 groups (*n* = 5). (**J**) IF staining of MECA-79 (green) in TDLN between 2 groups (*n* = 4). (**K**) FCM of CFSE^+^ cells in TDLN between the control and bevacizumab groups (*n* = 4). (**L**) FCM of LTβR expression on the surface of HEV between the control and bevacizumab groups (*n* = 3). (**M**) IHC staining of LYVE-1 expression in TDLN between the control and bevacizumab groups (*n* = 4). (**N**) IHC staining of CCL21 expression in TDLN between the control and bevacizumab groups (*n* = 3). Data are shown as mean ± SD (**B**–**E** and **G**–**N**). (**B**–**E** and **G**–**N**) *P* values were measured by unpaired, 2-tailed Student’s *t* test with or without Welch’s correction analysis. *P < 0.05, ***P < 0.001, ****P < 0.0001. Scale bars: 40 μm (**H** and **I**) and 200 μm (**J**).

Bevacizumab is a monoclonal antibody that targets VEGFA and is commonly used to inhibit VEGFA expression by specifically binding to VEGFA and blocking VEGF binding to VEGFR (17). We used bevacizumab to inhibit tumor-derived VEGFA production and explore the role of VEGFA in the PMN within the TDLN. We showed that bevacizumab substantially suppressed VEGFA protein expression in LLC cells (Supplemental Figure 3A). In addition, we found that the expression of VEGFA in the cell supernatant of the bevacizumab group was lower than that in the control group (Supplemental Figure 3B), and considering that the source of VEGFA in the cell supernatant was mainly the LLC cells, we concluded that bevacizumab could inhibit VEGFA secretion. In tumor-bearing mice treated with bevacizumab for 2 weeks (Figure 4A), we observed a marked reduction in tumor growth and delayed tumor progression (0.71 ± 0.26 g versus 0.36 ± 0.08 g) (Figure 4, B and C). We detected TDLN metastasis via H&E staining, and the results revealed that the LN metastasis rate in the bevacizumab group (66.7%, 4 of 6) was lower than that in the control group (33.3%, 2 of 6), suggesting that bevacizumab inhibited LN metastasis (Figure 4D). Previous studies reported that primary tumors can secrete VEGFA into the peripheral blood and promote angiogenesis (18); therefore, we examined the levels of VEGFA in the peripheral blood of the 2 groups, and the results show that the VEGFA level in the bevacizumab group was substantially lower than that in the control group (Figure 4E), suggesting that bevacizumab could inhibit VEGFA secretion. In the tumor tissue, both the VEGFA and VEGFR2 protein levels were reduced, whereas the VEGFR1 and VEGFR3 protein levels were not substantially changed (Supplemental Figure 3C). VEGFA expression in TDLNs was similarly reduced (Figure 4F), whereas its expression in NDLNs was not substantially different (Supplemental Figure 3D). These results indicate that tumor regulation of LNs follows a sequential pattern, highlighting the potential importance of TDLN dissection in cancer therapy (19). In addition, we measured the percentages of CD4^+^ and CD8^+^ T cells and DC cells in the control and bevacizumab groups, and the results were 20.01% versus 20.93%, 11.50% versus 13.10%, and 6.77% versus 7.01% (Figure 4G), respectively, which were not substantially different, suggesting that VEGFA may not be involved in the regulation of lymphocytes. We examined the expression of CD31to further explore the relationship between VEGFA and angiogenesis. We found that CD31 expression in tumor tissues (Figure 4H) and TDLNs was lower in the bevacizumab group than in the control group (Figure 4I), suggesting that VEGFA promoted angiogenesis in TDLNs. The results reveal no significant difference in the number of total HEVs (128.8 ± 21.6 versus 136.5 ± 30.9) or the number of dilated HEVs (58.0 ± 12.8 versus 63.3 ± 17.8) between the control and bevacizumab groups (Figure 4J). Moreover, CFSE^+^ cell counts (Figure 4K) and LTβR protein expression did not differ substantially between the 2 groups (Figure 4L), indicating that VEGFA was not involved in regulating HEV proliferation or function.

We elucidated other pathways by which VEGFA promotes PMN formation by examining the expression of the lymphatic vessel marker LYVE-1 in 2 groups of TDLNs. The results show lower LYVE-1 expression in the control group than in the bevacizumab group (Figure 4M), suggesting that VEGFA promoted lymphangiogenesis in TDLNs. CCL21 is a chemokine that regulates lymphocyte homing (20), and we confirmed that CCL21 expression was lower in the bevacizumab group than in the control group (Figure 4N). These results suggest that VEGFA is not associated with HEV dedifferentiation or lymphocyte homing but may be involved in the PMN through other pathways, such as lymphangiogenesis.

### VEGFC and VEGFD promote tumor cell migration.

We generated VEGFC-overexpressing (VEGFC-OE) and VEGFD-OE LLC cell lines to explore the roles of VEGFC and VEGFD in TDLNs. The results reveal that both the VEGFC protein and RNA levels were substantially elevated in the VEGFC-OE cell line compared with those in the control cells (Supplemental Figure 4, A and B), along with increased VEGFC secretion into the supernatant (Supplemental Figure 4C). Similarly, VEGFD protein and RNA levels (Supplemental Figure 4, D and E), as well as its secretion (Supplemental Figure 4F), were notably higher in the VEGFD-OE cell line. Transwell and scratch wound healing assays revealed that the migratory capacity of both VEGFC-OE (Supplemental Figure 4, G and H) and VEGFD-OE cells (Supplemental Figure 4, I and J) was substantially increased. These results suggest that VEGFC and VEGFD promote LLC migration and increase the metastatic capacity of LLC cells.

### Tumor-secreted VEGFC promotes HEV proliferation in TDLNs.

**Figure 5:**
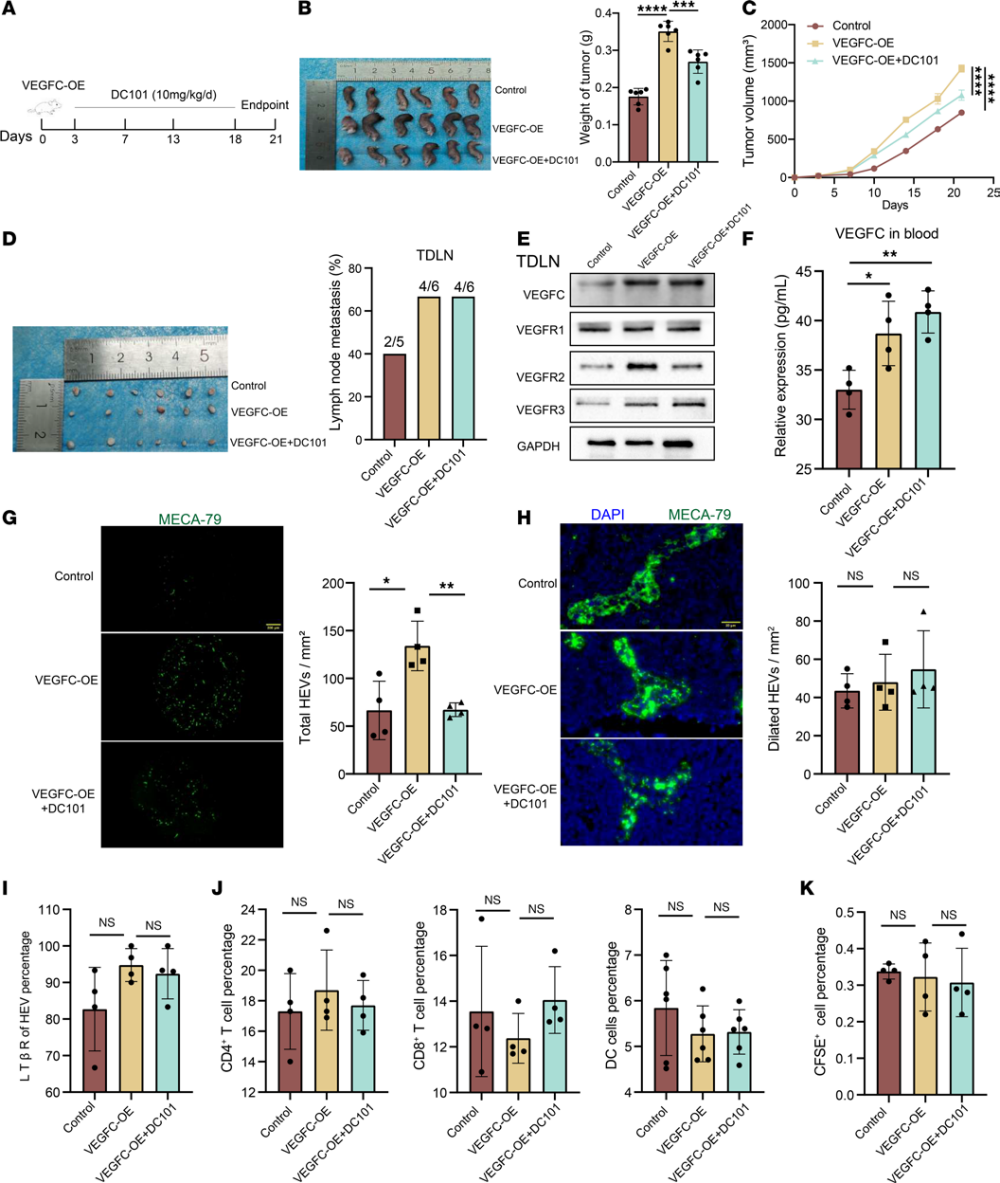
VEGFC promotes HEV proliferation in TDLN. (**A**) DC101 treatment protocol. (**B**) Tumor weight in control, VEGFC overexpressing (VEGFC-OE), and VEGFC-OE+DC101 groups (*n* = 6). (**C**) Tumor growth curves in control, VEGFC-OE, and VEGFC-OE+DC101 groups (*n* = 5). (**D**) Tumor draining lymph node (TDLN) metastasis rate in control (*n* = 5), VEGFC-OE (*n* = 6), and VEGFC-OE+DC101 groups (*n* = 6). (**E**) Western blotting (WB) assay of VEGFC and its related receptors expression in each group (*n* = 3). (**F**) ELISA of VEGFC expression in peripheral blood between 3 groups (*n* = 6). (**G**) Immunohistofluorescence (IF) staining for MECA-79 (green) was used to calculate the total number of high endothelial venules (HEVs) in each group (*n* = 4). (**H**) IF staining for MECA-79 (green) was used to calculate Dilated HEVs in each group (*n* = 4). (**I**) Flow cytometry (FCM) for LTβR expression on the surface of HEV in TDLN between control, VEGFC-OE, and VEGFC-OE+DC101 groups (*n* = 4). (**J**) FCM of CD4^+^ (*n* = 4) and CD8^+^ T cells (*n* = 4) and DCs (*n* = 6) in TDLN between control, VEGFC-OE, and VEGFC-OE+DC101 groups. (**K**) FCM of CFSE^+^ cells in TDLN between control, VEGFC-OE, and VEGFC-OE+DC101 groups (*n* = 4). Data are shown as mean ± SD (**B**–**D** and **F**–**K**). (**B**–**D** and **F**–**K**) *P* values were measured by 1-way ANOVA with Dunnett’s multiple-comparison test. *P < 0.05, **P < 0.01, ***P < 0.001, ****P < 0.0001. Scale bars: 200 μm (**G**) and 20 μm (**H**).

These results indicate that, for HEV regulation, VEGFR2 is more likely to bind to VEGF-related proteins than are VEGFR1 or VEGFR3 (Figure 2E). Therefore, in this part of the study, we chose to use the VEGFR2 inhibitor DC101 as a blocking agent to inhibit VEGFR2 expression in the VEGFC-OE and VEGFD-OE groups (21). We divided the mice into 3 groups, the control group, the VEGFC-OE group, and the VEGFC-OE+DC101 group; constructed TDLN models with LLC and VEGFC-OE LLC cells; and treated the mice in the VEGFC-OE group with DC101 (Figure 5A). The results show that the tumor weight in the VEGFC-OE group was substantially greater than that in the control group and that, in the VEGFC-OE+DC101 group (0.18 ± 0.02 g versus 0.36 ± 0.01 g versus 0.28 ± 0.02 g) (Figure 5B), the tumor volume was substantially greater than that in the control group and the VEGFC-OE+DC101 group (Figure 5C). H&E staining of TDLNs revealed that the LN metastasis rate in the control group (40.0%, 2 of 5) was lower than that in the VEGFC-OE group (66.7%, 4 of 6) and the VEGFC-OE+DC101 group (66.7%, 4 of 6) (Figure 5D). These results indicate that VEGFC can promote tumor progression and LN metastasis. We detected the protein expression of VEGFC and related receptors in the TDLNs from the 3 groups. The expression of VEGFC and VEGFR3 was higher in the VEGFC-OE group compared to the control group. The expression of VEGFR2 was lower after DC101 treatment, and no difference in the expression of VEGFR1 was observed (Figure 5E). We further examined the expression of VEGFC in tumors to clarify the reason for the elevated VEGFC levels in TDLNs, and the results reveal that the expression of VEGFC, VEGFR1, and VEGFR3 in tumors from the VEGFC-OE group was increased and that the expression of VEGFR2 was decreased in the VEGFC-OE group compared with the VEGFC-OE+DC101 group (Supplemental Figure 5A). The results confirm that the trend of VGEFC expression was consistent in TDLNs and in tumors. We examined the expression of VEGFC in peripheral blood to explore the regulatory effect of the primary tumor on the TDLNs, and the results reveal that VEGFC expression in the VEGFC-OE group was higher than that in the control group (Figure 5F), which suggested that the primary tumor may secrete VEGFC into the TDLN through the peripheral blood to increase VEGFC expression in the TDLN.

Furthermore, we explored the morphological and functional characteristics of HEVs, and the results show that the numbers of total HEVs in the control, VEGFC-OE, and VEGFC-OE+DC101 groups were 66.5 ± 30.6, 134.0 ± 25.9, and 67.3 ± 7.1, respectively, and the number of HEVs in the VEGFC-OE group was substantially greater than that in the control group but lower than that in the VEGFC-OE+DC101 group (Figure 5G). The numbers of dilated HEVs were 43.5 ± 8.9, 48.0 ± 14.7, and 54.8 ± 20.2 in each group, with no significant differences (Figure 5H). LTβR expression was also not substantially different among the 3 groups in TDLNs (Figure 5I) or NDLNs (Supplemental Figure 5B). This finding suggests that VEGFC/VEGFR2 promotes HEV proliferation but does not affect morphology. Therefore, is lymphocyte homing affected accordingly? We examined the differences in the proportions of lymphocytes in TDLNs among the different groups and found that the proportions of CD4^+^ T cells, CD8^+^ T cells, and DC cells in the VEGFC-OE group were not substantially different from those in the control group and VEGFC-OE + DC101 group (Figure 5J). Similarly, no statistically significant differences were observed in the NDLNs (Supplemental Figure 5, C and D). We found that the percentages of CFSE^+^ cells in the control group, VEGFC-OE group, and VEGFC-OE+DC101 group were 0.34%, 0.32%, and 0.31%, respectively, and no statistically significant difference was observed between the groups (Figure 5K). The proportion of CFSE^+^ cells in the NDLNs of each group also did not differ substantially (Supplemental Figure 5E). We speculated that lymphocyte homing may be closely related to the morphology of HEVs, as VEGFC/VEGFR2 promotes an increase in the number of HEVs, but their lymphocytes are not affected.

### VEGFD induces HEV dedifferentiation in TDLNs.

**Figure 6:**
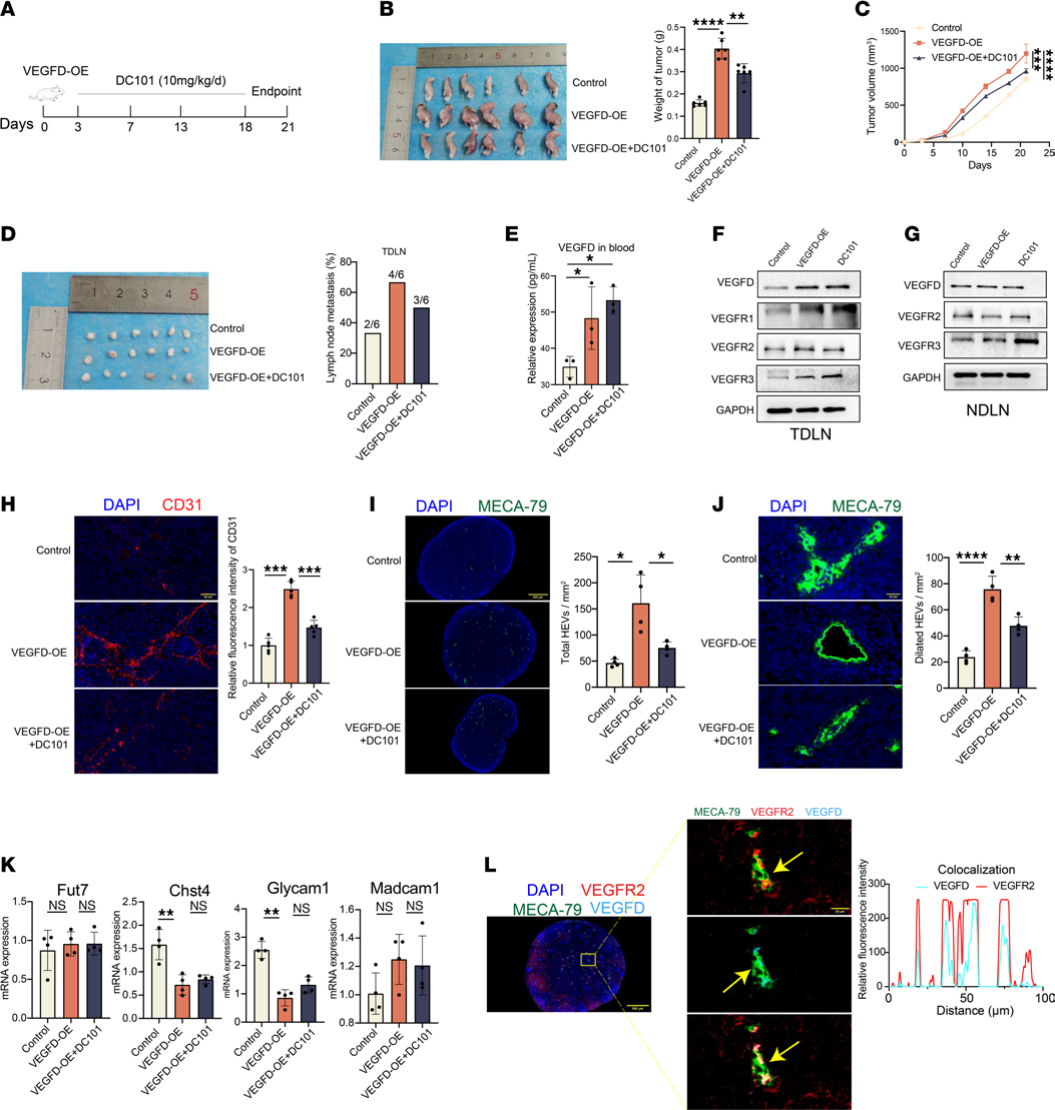
VEGFD induces HEV dilation in TDLN. (**A**) DC101 treatment protocol. (**B**) Tumor weight in control, VEGFD overexpressing (VEGFD-OE), and VEGFD-OE+DC101 groups (*n* = 6). (**C**) Tumor growth curves in control, VEGFD-OE, and VEGFD-OE+DC101 groups (*n* = 5). (**D**) Tumor draining lymph node (TDLN) metastasis rate in control, VEGFD-OE, and VEGFD-OE+DC101 groups (*n* = 6). (**E**) ELISA of VEGFD expression in peripheral blood (*n* = 3). (**F**) Western blotting (WB) assay of VEGFD and its related receptors expression in TDLN between control, VEGFD-OE, and VEGFD-OE+DC101 groups (*n* = 3). (**G**) WB assay of VEGFD expression and its related receptors in nondraining lymph node (NDLN) between control, VEGFD-OE, and VEGFD-OE+DC101 groups (*n* = 3). (**H**) Immunohistofluorescence (IF) staining of CD31 (red) expression in TDLN between control, VEGFD-OE, and VEGFD-OE+DC101 groups (*n* = 4). (**I**) IF staining of MECA-79 (green) to count total high endothelial venules (HEVs) in each group (*n* = 4). (**J**) IF staining of MECA-79 (green) to count dilated HEVs in each group (*n* = 4). (**K**) qPCR for the HEV markers (Fut7, Chst4, Glycam1, and Madcam1) in TDLN between control, VEGFD-OE, and VEGFD-OE+DC101 groups (*n* = 4). (**L**) IF staining for MECA-79 (green), VEGFR2 (red), and VEGFD (blue) coexpression. Data are shown as mean ± SD (**B**–**E** and **H**–**K**). (**B**–**E** and **H**–**K**) *P* value measured by one-way ANOVA with Dunnett’s multiple-comparison test. *P < 0.05, **P < 0.01, ***P < 0.001, ****P < 0.0001, ns: no significant. Yellow arrows, colocalization of HEV, VEGFD, and VEGFR2. Scale bars: 40 μm (**H**), 500 μm (**I** and **L**) and 20 μm (**J** and **L**).

Similarly, we constructed a TDLN model using control and VEGFD-OE LLC cells to elucidate the effect of VEGFD on HEVs, and after the overexpression of VEGFD, we blocked the expression of the VEGFR2 protein with DC101 (Figure 6A). We randomly divided the mice into 3 groups: the control, VEGFD-OE, and VEGFD-OE+DC101 groups. The results reveal that the tumor weights of the control, VEGFD-OE, and VEGFD-OE+DC101 groups were 0.16 ± 0.01 g, 0.40 ± 0.05 g, and 0.29 ± 0.04 g, respectively (Figure 6B). The tumor weights and volumes in the VEGFD-OE group were larger than those in the control group and in the VEGFD-OE+DC101 group (Figure 6C). H&E staining of TDLNs revealed a higher LN metastasis rate in the VEGFD-OE group (66.7%, 4 of 6) than in the control group (33.3%, 2 of 6) and a lower rate than that in the VEGFC-OE+DC101 group (50.0%, 3 of 6) (Figure 6D). These results suggest that VEGFD promotes tumor progression and LN metastasis. Furthermore, we assayed VEGFD expression in the 3 groups. We found that, in peripheral blood, VEGFD expression was substantially higher in the VEGFD-OE and VEGFD-OE+DC101 groups than in the control group (Figure 6E). According to the above description, primary tumors may induce elevated TDLN expression through the secretion of VEGFD in the peripheral blood, and we examined the expression of VEGFD and VEGF-related receptors in the TDLNs and NDLNs. The results from TDLNs showed that the expression of VEGFD, VEGFR1, and VEGFR3 was higher in the VEGFD-OE and VEGFD-OE+DC101 groups than in the control group, and the expression of VEGFR2 was lower in the VEGFD-OE+DC101 group than in the VEGFD-OE group (Figure 6F), possibly because of DC101 treatment. In the NDLNs, VEGFD and VEGFR2 expression did not differ substantially among the control and VEGFD-OE groups, and VEGFR3 expression was higher in the VEGFD-OE+DC101 group than in the VEGFD-OE group (Figure 6G). We concluded that the primary tumor induced high VEGFD expression in the TDLNs but had no significant effect on the NDLNs, which may be determined by the anatomical location.

We confirmed the increased expression of VEGFD in TDLNs from the VEGFD-OE group; therefore, we tested the relevant indices of HEVs to determine whether it affects HEVs. We examined CD31 expression in TDLNs from each group and found that CD31 expression was higher in the VEGFD-OE group than in the control group but lower than in the VEGFD-OE+DC101 group, suggesting that the VEGFD/VEGFR2 axis induced angiogenesis in TDLNs (Figure 6H). We further counted the number of HEVs and the number of dilated HEVs in each group, and the results reveal that the total number of HEVs in the VEGFD-OE group (161.0 ± 54.2) was greater than that in the control group (46.5 ± 7.3) but less than that in the VEGFD-OE+DC101 group (75.3 ± 11.6) (Figure 6I). Similarly, the number of dilated HEVs in the VEGFD-OE group (23.8 ± 4.9) was greater than that in the control group (75.6 ± 10.1) but less than that in the VEGFD-OE+ DC101 group (47.8 ± 6.8) (Figure 6J), suggesting that VEGFD/VEGFR2 signaling increased the number of HEVs and HEV expansion. We examined the expression of HEV markers and found that the expression of Chst4 and Glycam1 in the VEGFD-OE group was lower than that in the control group and was not substantially different from that in the VEGFD-OE+DC101 group; we also examined the expression of Fut7 and Macadm1 and found that it was not substantially different among the groups (Figure 6K). We examined the coexpression of HEV, VEGFR2, and VEGFD to explore how VEGFD binds to VEGFR2. The results reveal a correlation between VEGFD and VEGFR2 expression on the surface of HEVs (Figure 6L), which suggests that VEGFD may bind to VEGFR2 on the surface of HEVs and induce several changes in HEVs.

In addition, we explored the associations of VEGFD with HEVs and lymphocyte homing in a melanoma model. We constructed VEGFD-OE B16-F10 cells and showed that VEGFD protein and mRNA expression were substantially increased in VEGFD-OE cells (Supplemental Figure 6A). The results show that, in the VEGFD-OE group, the tumor weight (Supplemental Figure 6B) and volume (Supplemental Figure 6C) were substantially greater than those in the control group, which indicated that VEGFD could substantially promote melanoma progression. H&E staining showed that the TDLN metastasis rate in the VEGFD-OE group (4 of 6) was higher than that in the control group (2 of 6) (Supplemental Figure 6D). We examined the differences in the number of HEVs in the TDLNs from each group and found that the number of dilated HEVs was greater in the VEGFD-OE group (67.25 ± 7.93) than in the control group (28.75 ± 4.65) (Supplemental Figure 6E). The results of the flow cytometry (FCM) assay indicated that the expression of LTβR on the surface of HEVs was lower in the VEGFD-OE group than in the control group (Supplemental Figure 6F), suggesting that VEGFD also induced HEV dedifferentiation in melanoma. We examined the lymphocyte content in TDLNs to clarify whether HEV-mediated lymphocyte homing was impaired, and the results indicate that the percentages of CD4^+^ T cells (19.9% versus 13.1%) and CD8^+^ T cells (14.0% versus 8.6%) in the VEGFD-OE group were substantially lower than those in the control group (Supplemental Figure 6G). The percentages of CFSE^+^ cells in the control and VEGFD-OE groups were 1.28% and 0.51%, respectively (Supplemental Figure 6H), and the number of CFSE^+^ cells in the VEGFD-OE group was lower than that in the control group. These results indicate that VEGFD can also induce HEV dedifferentiation to impair lymphocyte homing to TDLNs in patients with melanoma.

### VEGFD knockdown promotes HEV maturation and lymphocyte homing.

**Figure 7:**
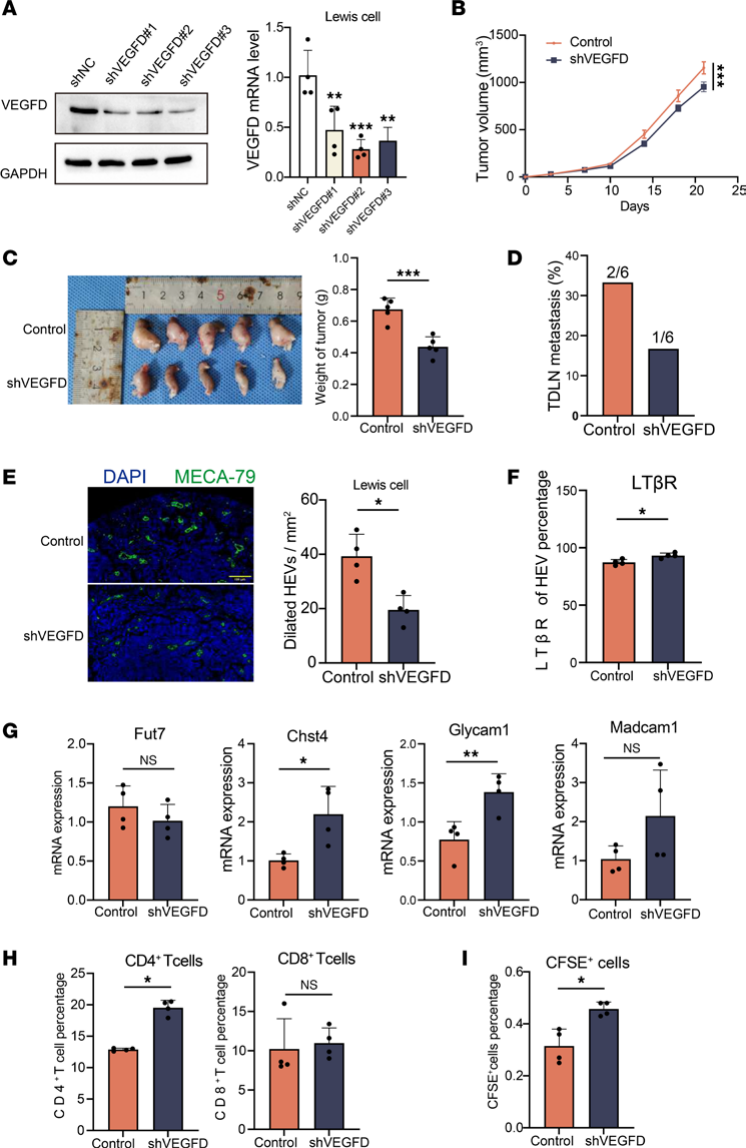
Knockdown of VEGFD promotes HEV maturation and lymphocyte homing. (**A**) Western blotting and qPCR were performed to detect VEGFD expression in Lewis cells. (**B**) Tumor growth curves in the control and shVEGFD groups (*n* = 5). (**C**) Tumor weight in the control and shVEGFD groups (*n* = 5). (**D**) Tumor draining lymph node (TDLN) metastasis rate in the control and shVEGFD groups (*n* = 6). (**E**) Immunohistofluorescence staining of MECA-79 (green) in TDLN between 2 groups (*n* = 4). (**F**) Flow cytometry (FCM) of LTβR expression on the surface of HEV between the control and shVEGFD groups (*n* = 4). (**G**) qPCR for the HEV markers (Fut7, Chst4, Glycam1, and Madcam1) in TDLN between control and shVEGFD groups (*n* = 4). (**H**) The percentage of CD4^+^ and CD8^+^T cells in TDLN between control and shVEGFD groups by FCM (*n* = 4). (**I**) The percentage of CFSE^+^ cells in TDLN by FCM (*n* = 4). In this figure, data are shown as mean ± SD. (**A**) *P* value measured by 1-way ANOVA with Dunnett’s multiple-comparison test. (**B**–**I**) *P* values were measured by unpaired, 2-tailed Student’s *t* test with or without Welch’s correction analysis. *P < 0.05, **P < 0.01, ***P < 0.001. Scale bar: 100 μm (**E**).

We explored whether VEGFD knockdown results in a phenotype opposite to that resulting from VEGFD overexpression by constructing VEGFD-knockdown LLC cells. The results reveal substantially lower VEGFD protein and RNA expression in the shVEGFD group than that in the shNC group (Figure 7A), indicating successful shVEGFD-transfected LLC cell construction. We found that the tumor volume (Figure 7B) and weight (0.44 ± 0.06 g versus 0.67 ± 0.07 g) in the shVEGFD group (Figure 7C) were substantially lower than those in the control group. Furthermore, H&E staining showed that the TDLN metastasis rate in the shVEGFD group was 16.7% (1 of 6), which was lower than that in the control group (33.3%, 2 of 6) (Figure 7D), suggesting that lowering VEGFD expression could inhibit tumor growth and LN metastasis. We examined the number of dilated HEVs in each group, and the proportion of dilated HEVs was substantially lower in the shVEGFD group than in the control group (Figure 7E). We detected the expression of LTβR, an important marker for maintaining the maturation characteristics of HEVs, and the results reveal that the expression of LTβR was higher in the shVEGFD group than in the control group (Figure 7F). In addition, we detected other HEV markers and found that the expression of Chst4 and Glycam1 was higher in the shVEGFD group than in the control group, but significant differences in the expression of Fut7 and Macadm1 were not observed (Figure 7G). These results suggest that the inhibition of VEGFD expression can restore the maturation state of HEVs. We examined the content of lymphocytes in TDLNs in each group and found that the proportion of CD4^+^ T cells in the shVEGFD group was substantially higher than that in the control group (19.9% versus 12.9%), whereas no significant difference in the proportion of CD8^+^ T cells was observed (10.9% versus 10.2%) (Figure 7H). In addition, the proportion of CFSE^+^ cells in the shVEGFD group was higher than that in the control group (0.46% versus 0.32%) (Figure 7I). These results suggest that the knockdown of VEGFD substantially improves HEV-mediated lymphocyte homing.

### The VEGFD/VEGFR2 axis regulates HEV-mediated lymphocyte homing.

**Figure 8:**
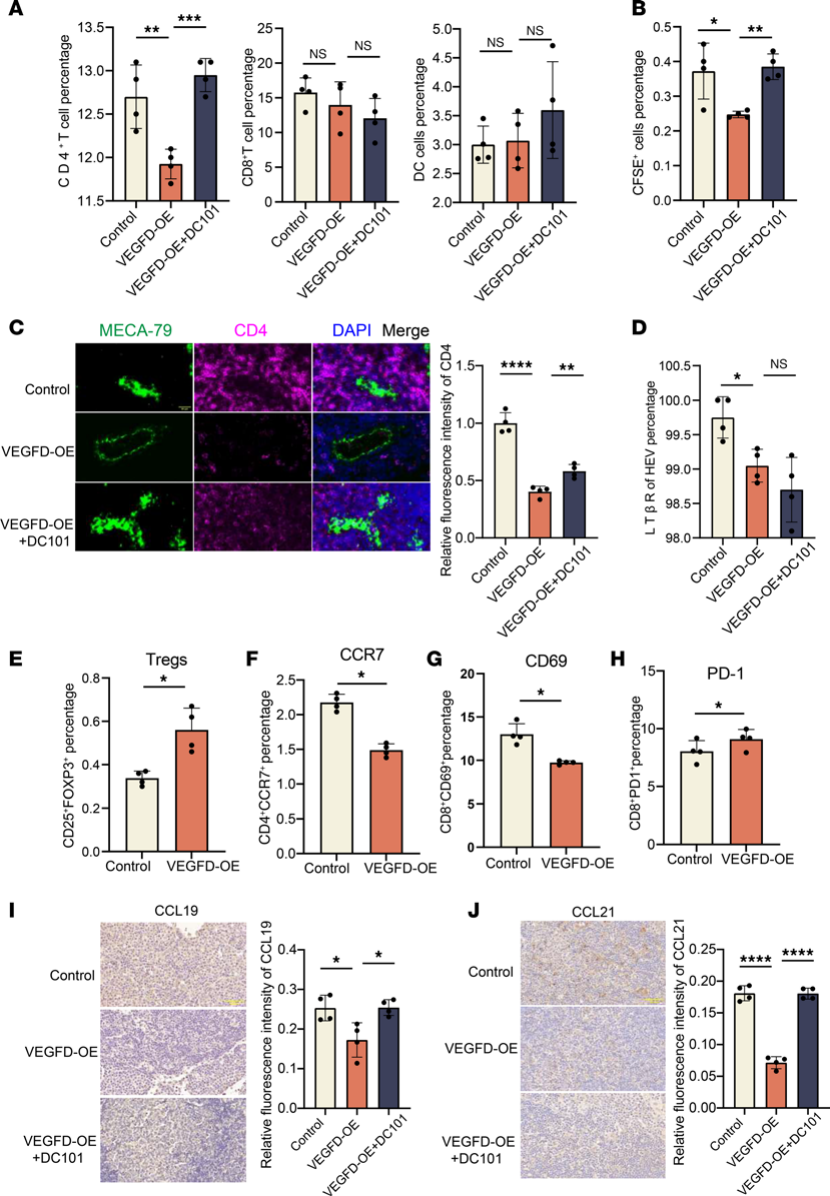
VEGFD/VEGFR2 axis regulates lymphocyte homing of HEV. (**A**) The percentage of CD4^+^ and CD8^+^ T cells and DCs in tumor draining lymph node (TDLN) between control, VEGFD-OE, and DC101 groups by flow cytometry (FCM) (*n* = 4). (**B**) The percentage of CFSE^+^ cells in TDLN by FCM (**C**) (*n* = 4). (**C**) Immunohistofluorescence staining for CD4 (pink) and MECA-79 (green) in each group (*n* = 4). (**D**) FCM of LTβR expression on the surface of high endothelial venule (HEV) in TDLN between 3 groups (*n* = 4). (**E**) FCM of Tregs in control and VEGFD-OE groups (*n* = 4). (**F**) FCM of CD4^+^CCR7^+^ in control and VEGFD-OE groups (*n* = 4). (**G**) FCM of CD8^+^CD69^+^ in control and VEGFD-OE groups (*n* = 4). (**H**) FCM of CD8^+^PD1^+^ in control and VEGFD-OE groups (*n* = 4). (**I**) IHC of CCL19 expression in each group (*n* = 4). (**J**) IHC of CCL21 expression in each group (*n* = 4). In this figure, data are shown as mean ± SD. (**A**–**D**, **I**, and **J**) *P* value measured by 1-way ANOVA with Dunnett’s multiple-comparison test. (**E**–**H**) *P* values were measured by unpaired, 2-tailed Student’s *t* test with or without Welch’s correction analysis. *P < 0.05, **P < 0.01, ***P < 0.001, ****P < 0.0001. Scale bars: 20 μm (**C**) and 50 μm (**I** and **J**).

We clarified the function of VEGFD/VEGFR2 in lymphocyte homing by counting the number of lymphocytes in each group. In TDLNs, the proportion of CD4^+^ T cells in the VEGFD-OE group (11.9%) was lower than that in the control group (12.7%) and the VEGFD-OE+DC101 group (13.0%), the proportion of CD8^+^ T cells in the VEGFD-OE group (13.9%) was lower than that in the control group (15.7%) but not was substantially different, and a significant difference in the proportion of CD8^+^ T cells was not observed after DC101 treatment (12.1%); moreover, the proportion of DCs was not substantially different among the groups (3.0%, 3.1%, and 3.6%, respectively) (Figure 8A). In addition, we measured the proportion of CFSE^+^ cells in TDLNs and found that the percentage of CFSE^+^ cells in the VEGFD-OE group (0.24%) was lower than that in the control group (0.37%) and the VEGFD-OE+DC101 group (0.38%) (Figure 8B), suggesting that HEV-mediated lymphocyte homing was impaired in the VEGFD-OE group. We found that the total CD4^+^ T cell content of TDLNs was affected by the VEGFD/VEGFR2 axis. Thus, we questioned whether this effect could be caused by impaired HEV-mediated CD4^+^ T cell homing. We therefore examined CD4^+^ T cell expression around HEVs and observed lower CD4 expression in the VEGFD-OE group than that in the control group and the VEGFD-OE+DC101 group (Figure 8C), suggesting that VEGFD may have impaired CD4^+^ T cell homing, which in turn led to a decrease in the proportion of CD4^+^ T cells in the TDLNs. We found that the expression of LTβR on the surface of HEVs in the VEGFD-OE group (99.05%) was lower than that in the control group (99.75%) and was not substantially different from that in the VEGFD-OE+DC101 group (98.70%) (Figure 8D). The above results suggest that VEGFD can lead to a decrease in the number of T cells.

Furthermore, does VEGFD affect the function of T cells? We examined the number of Tregs expressing CCR7, CD69, and PD1 via FCM, and the results indicate that the proportion of Tregs in the VEGFD-OE group (0.55%) was higher than that in the control group (0.34%) (Figure 8E), and the expression of CCR7 on CD4^+^ T cells in the VEGFD-OE group (2.2%) was lower than that in the control group (1.5%) (Figure 8F). CD69 and PD1 are markers of CD8^+^ T cell activation and inhibition (22). We detected lower expression of CD69 in the VEGFD-OE group (9.8%) than in the control group (12.8%) (Figure 8G), and PD1 expression in the VEGFD-OE group (9.1%) was lower than that in the control group (8.0%) (Figure 8H), which indicated that VEGFD not only decreased the number of T cells but also inhibited the function of T cells. We elucidated the cause of impaired CD4^+^ T cell homing by measuring the expression of chemokines expressed on lymphocytes, and the results show that the expression of CCL19 (Figure 8I) and CCL21 (Figure 8J) in the VEGFD-OE group was lower than that in the control group and the VEGFD-OE+DC101 group, suggesting that VEGFD may reduce the expression of CCL19 and CCL21 and lead to impaired CD4^+^ T cell homing.

## Discussion

In this study, we confirmed the intricate interactions between VEGF signaling, HEVs, and lymphocytes in TDLNs, showing that VEGFA, VEGFC, and VEGFD play distinct roles in shaping the PMN. While previous studies have focused primarily on the role of VEGF signaling in tumor cells (23), direct evidence for its influence on TDLNs is limited. We found that VEGF-related proteins are substantially upregulated in TDLNs, directly altering the LN microenvironment and contributing to PMN formation. To the best of our knowledge, this study is the first to report that the binding of VEGFD to VEGFR2 in the TDLNs directly leads to the HEV-mediated impairment of lymphocyte homing. This disruption in lymphocyte trafficking subsequently contributes to compromised immune responses in the TDLNs, thereby promoting the formation of the PMN. These findings reveal a potentially novel mechanism by which VEGF-related signaling in TDLNs facilitates immune evasion and sets the stage for tumor metastasis.

VEGFA, VEGFC, and VEGFD play pivotal roles in promoting LN metastasis by enhancing lymphangiogenesis and vascular remodeling (24–26). These investigations, however, predominantly focused on the alterations occurring within the tumor tissues while usually overlooking changes in the TDLN microenvironment. As crucial immune organs, LNs are central to orchestrating immune responses (27). In our study, we observed significant reductions in the numbers of CD4^+^ and CD8^+^ T cells and DCs in the TDLNs compared with those in the NDLNs and NLNs. Since these lymphocytes are vital for initiating immune responses, their depletion within TDLNs may severely impair the capacity of the LNs to mount an effective defense against invading tumor cells. The increased recruitment of immunosuppressive cells, such as Tregs and MDSCs, exacerbates immune suppression, promoting immune evasion and distant organ metastasis (28). We found that VEGFD induced a suppressive immune microenvironment in the TDLNs through multiple pathways. First, VEGFD impaired HEV-mediated T lymphocyte homing, resulting in a decrease in the number of CD4^+^ T cells that homed to the TDLNs; furthermore, VEGFD increased in the number of immunosuppressive cells (Tregs and CD8^+^ PD1^+^ T cells) and decreased the expression of CD69, which suppressed the activation of T cells. These changes likely foster a permissive environment, facilitating metastatic spread. Wakisaka et al. observed that, in human oral squamous carcinoma samples, the expression of VEGFD in primary tumors is independent of the HEV density and that HEVs are not involved in lymph node metastasis (29), which seem to contradict our findings. This discrepancy may be due to the use of human and mouse models, where HEVs are more susceptible to the regulatory effects of VEGF in mouse TDLNs, and this observation has also been confirmed in CNE2 tumor model mice (15). In addition, oral squamous cell carcinoma and lung cancer are different tumor types, which may also account for the differences in the results.

HEVs play a crucial role in facilitating lymphocyte homing in LNs. In this study, we identified the significant expansion and dedifferentiation of HEVs in TDLNs, which aligns with findings from earlier research (15, 30). However, while previous studies have focused predominantly on structural changes in HEVs, they lack functional analyses. By employing lymphocyte homing assays, we demonstrated that the dedifferentiation of HEVs is an associated factor leading to the depletion of immune cells in TDLNs. The mechanisms underlying HEV dedifferentiation are complex and not yet fully elucidated. In our study, we observed that the expression of LTβR, a key signal maintaining HEV stability (31), was reduced on the surface of dedifferentiated HEVs, yet, overall, LTβR signaling in TDLNs was increased. This result may indicate increased LTβR expression in other cell types in LNs, compensating for its reduction in HEVs.

Furthermore, we detected significant changes in the levels of key HEV markers, including Fut7, Glycam1, Chst4, and Macadm1. Interestingly, under the regulation of VEGFD, only Chst4 and Glycam1 expression were notably affected, suggesting that these markers may be governed by distinct regulatory mechanisms. Solid stress is the pressure generated by tumor cells and the extracellular matrix (32). Jones et al. found that solid stress induced vascular remodeling and downregulated the expression of the HEV signature genes Chst4 and Fut7, which further impaired T cell homing function (33). We observed that VEGFD induced TDLN angiogenesis and HEV dedifferentiation, but whether these effects are related to solid stress needs to be confirmed by further studies. Further research is necessary to fully understand the signaling networks involved in HEV dedifferentiation and their implications for the TDLN microenvironment.

Mechanistically, CD4^+^ T cell entry into LNs appears to be modulated by the dedifferentiation of HEVs, which is mediated by the VEGFD/VEGFR2 axis. Although both CD4^+^ and CD8^+^ T cells are T cells, their homing mechanisms are not identical. HIV-1Nef impairs the extravasation and homing of CD4^+^ T cells (34), and Lupsa et al. reported that peptidase inhibitor 16 was associated with the homing of CD8^+^ T cells in skin tissue (35). Therefore, we conclude that VEGFD/VEGFR2-mediated HEV dedifferentiation affects the homing of CD4^+^ T cells but not that of CD8^+^ T cells. In healthy LNs, HEVs are critical for facilitating lymphocyte entry by expressing specific adhesion molecules and chemokines, such as CCL19 and CCL21, which guide lymphocytes to their respective regions in the LNs (36). Furthermore, we hypothesize that different lymphocyte subtypes utilize distinct homing mechanisms, with the lymphatic system also contributing to the homing of a subset of lymphocytes (37). Notably, VEGFA promotes lymphangiogenesis in TDLNs, while the expression of the chemokine CCL21 is reduced. The VEGFA-induced remodeling of lymphatic vessels may contribute to PMN establishment, independent of traditional lymphocyte homing pathways, potentially promoting metastatic progression. Our study examined lymphocyte homing times of 1–2 hours, which is the time period that most studies have focused on (38, 39), and confirmed a substantial difference; however, whether longer times generate different results requires further study.

The VEGFD/VEGFR2 axis is a crucial signaling pathway that governs tumor angiogenesis (40–42). In this study, we elucidated a potentially novel mechanism in which the VEGFD/VEGFR2 axis regulates the dedifferentiation of HEVs and impairs lymphocyte homing functions in TDLNs. This finding is particularly important for the prevention of LN metastasis, as it highlights a key pathway that compromises immune surveillance by reducing lymphocyte infiltration into the LNs, a process critical for controlling tumor spread (43). Interestingly, we found that the VEGFD/VEGFR2 axis induced lumen expansion in HEVs and did not upregulate LTβR expression after DC101 treatment. LTβR is an important molecule for maintaining HEV homeostasis; however, HEV dedifferentiation involves complex mechanisms, including CD11c^+^ DCs and the expression of LTα and LTβ (44, 45). Similarly, we found that the expression of the HEV markers Chst4, Fut7, and Glycam1 was not entirely consistent; thus, we hypothesized that HEV dedifferentiation involves the regulation of multiple mechanisms and that VEGFD/VEGFR2 may regulate HEV function via a pathway that is not dependent on LTβR. Further investigation into this axis could lead to novel interventions that not only block angiogenesis but also reinvigorate immune surveillance in TDLNs, a critical step in preventing the early stages of metastasis (46).

Overall, the mechanisms by which VEGFA/C/D promote LN metastasis are not identical, and we show that VEGFD/VEGFR2 signaling induced HEV dedifferentiation in TDLNs to impair CD4^+^ T cell homing and promote LN metastasis, which provides potential insights into the prevention of LN metastasis. 

## Methods

### Sex as a biological variable.

In this study, sex was not considered as a biological variable. We examined lymph node specimens from both male and female patients, with consistent findings observed across both groups. Only male animals were used in our experiments, as lung cancer has a higher reported incidence in males compared with females (47). Nonetheless, we believe our results are applicable to both male and female individuals.

### Cell lines.

We used human-derived lung adenocarcinoma cells (A549) and mouse LLC cells in this study. The LLC, A549, and B16-F10 cell lines were purchased from the Cell Bank of the Chinese Academy of Sciences (SCSP-5252, SCSP-503 and SCSP-5233). The LLC, A549, and B16-F10 cell lines were cultured in DMEM (Solarbio) supplemented with 10% FBS (Pricella).

### TDLN mouse model.

BALB/c nude (male, 4–6 weeks old) and C57BL/6J mice (male, 6–8 weeks old) were purchased from GemPharmatec. All the mice were housed in a specific pathogen–free (SPF) environment with adequate food and water. Cells in the logarithmic growth phase were digested with trypsin and processed into a single-cell suspension, and the concentration was adjusted to 1 × 10^7^ to 5 × 10^7^ cells/mL. The right foot pad of each mouse was selected as the injection site, with 50–100 μL of cell suspension injected per mouse, and the number of cells inoculated per mouse was approximately 1 × 10^6^ to 5 × 10^6^ cells. Based on the anatomic location, the right popliteal lymph node was considered the TDLN, the ILN was considered the second station of the TDLN, the left popliteal lymph node was considered the NDLN, and the right popliteal lymph node of normal mice was defined as the NLN (48). The tumor or TDLN volume formula was calculated as follows: V (mm^3^) = 1/2 × length × width^2^.

### Human sample.

LN and lung cancer tissues were obtained from postoperative specimens obtained from patients at the Second Affiliated Hospital of Nanchang University. The samples were classified according to whether the LN was metastatic, as follows: (a) human NLN-LN samples from patients without tumors or other diseases; (b) NMLN samples from patients with lung cancer; and (c) MLN samples from patients with lung cancer.

### Drugs protocol.

Bevacizumab was provided by Roche Pharmaceuticals. The VEGFR2 inhibitor DC101 was purchased from BioXCell (catalog BE0060). The treatment protocol was as follows: i.p. injection of 10 mg/kg bevacizumab per mouse, which was administered twice weekly for 2–3 weeks. DC101 was administered by i.p. injection at a dose of 10 mg/kg per mouse, 3 times per week for 2–3 weeks (21).

### Constructing stable cell lines.

VEGFC and VEGFD lentiviral vectors were purchased from HANBIO. Lentiviral vectors and polybrene (HANBIO, HB-PB) were added when the cells grew to 50%–60% confluence, and the culture medium was changed to fresh medium after 48 hours. Subsequently, 1 μg/mL puromycin (HANBIO, HB-PU) was added, and the cells were cultured for 2–3 days. Quantitative PCR (qPCR) and Western blotting were used to detect the transfection efficiency.

### qPCR analysis.

We used Monzol Reagent Pro (Monad, MI20201S) to extract total RNA from cells and animal tissues. After the removal of the genomic DNA, RT Premix (TaKaRa, RR092S) was used to reverse transcribe the RNA into complementary DNA. TB Green Premix (TaKaRa, RR092A) was used for amplification reactions. The gene expression levels were quantified via the 2^–ΔΔCT^ method. The sequences of primers used in this study are shown in Supplemental Table 2.

### Western blotting.

Tissues and cells were lysed with radioimmunoprecipitation assay (RIPA) lysis buffer (Solarbio, R0020) and then incubated at 4°C for 30 minutes to allow the samples to be fully lysed. The supernatant was subsequently collected by centrifugation at 13,400*g* for 15 minutes for subsequent experiments. The protein concentration was determined using a bicinchoninic acid (BCA) protein assay kit (Beyotime, P0012), and the extracted protein samples were adjusted to equal concentrations. The samples were then mixed with loading buffer (Beyotime, P0015L) and boiled for 10 minutes to fully denature the proteins. Proteins were separated using a 10% gel (Epizyme, PG112) and transferred onto a polyvinylidene fluoride (PVDF) membrane. The PVDF membranes were blocked with 5% nonfat milk for 1 hour at room temperature. The details of the antibodies used in this study are shown in Supplemental Table 3. After blocking, the samples were incubated with the primary antibody at 4°C on a shaker overnight. Following 3 washes with TBST, the membrane was incubated with goat anti–rabbit or –mouse IgG (1:1,0000) (BOSTER, BA1039 and BA1056, respectively) at room temperature for 2 hours. The membrane was then washed 3 more times with TBST to remove any remaining secondary antibody. Enhanced chemiluminescence (ECL) substrate (Proteintech, PK10001) was applied, and the chemiluminescent signal was detected. Fiji software was used to quantify the grayscale values for each group.

### IHC staining.

LN tissues were fixed with 4% paraformaldehyde and embedded in paraffin to create 4 μm–thick sections. The sections were deparaffinized and blocked with goat serum at 37°C for 1 hour. After the blocking solution was removed, a prediluted primary antibody mixture was applied, and the sections were incubated overnight at 4°C (details of the primary antibodies are provided in Supplemental Table 3). After 3 washes with PBS, the sections were incubated with fluorescent secondary antibodies at 37°C for 2 hours in the dark. The sections were then washed 3 more times with PBS, followed by DAPI staining of the nuclei at room temperature for 5 minutes in the dark. After a final round of PBS washes, the sections were mounted with antifade mounting medium and visualized under a fluorescence microscope for imaging.

### Immunohistofluorescence staining.

LN tissues were fixed with 4% paraformaldehyde and embedded in paraffin to create 4 μm–thick sections. The sections were deparaffinized and blocked with goat serum at 37°C for 1 hour. After the blocking solution was removed, a prediluted primary antibody mixture was applied, and the sections were incubated overnight at 4°C (details of the primary antibodies are provided in Supplemental Table 3). After 3 washes with PBS, the sections were incubated with fluorescent secondary antibodies at 37°C for 2 hours in the dark. The sections were then washed 3 more times with PBS, followed by DAPI staining of the nuclei at room temperature for 5 minutes in the dark. After a final round of PBS washes, the sections were mounted with antifade mounting medium and visualized under a fluorescence microscope for imaging.

### Analysis of IHC and Immunohistofluorescence data.

We used Fiji software to count the expression of proteins bound to antibodies against lymphotoxin β receptor (LTβR), VEGFR1, VEGFR2, VEGFR3, CD31, CD4, etc. CaseViewer software was used to capture the images, and 3–5 fields of view were randomly selected and imaged. The expression of the protein bound to the target antibody in each field of view was calculated as follows: mean gray value = integrated density/area. The relative fluorescence intensity of each group was then calculated according to the mean gray value (49).

### HEV morphological characterization.

Definition of HEV dedifferentiation. Based on Ulvmar and Qian et al.’s description of the morphological features of HEV dedifferentiation (15, 50), we defined the criteria for HEV dedifferentiation in this study as follows: (a) the longest diameter of the HEV lumen >10 μm was defined as dilated; and (b) the morphology of the HEV appeared to be lacking in stereotyped endothelial cells and discontinuous MECA-79 expression. Dedifferentiated HEVs were judged to be in compliance with one of these criteria. Fiji software was used to measure the HEV lumen diameter, and each measurement was repeated no less than 3 times to calculate the number of expanded HEVs in each sample. Quantitative analysis of HEVs: MECA-79^+^ areas of LNs were collected in CaseViewer, followed by calculating the number of MECA-79^+^ cells using Fiji software, adjusting the H threshold, and calculating the number of positive cells in each sample, with no less than 3 measurements per sample (51).

### LN H&E staining.

H&E staining was performed to detect LN metastasis. LNs were harvested from the mice. LNs were fixed with formalin, embedded in paraffin, and sectioned into 5–10 μm slices. The tissue sections were stained with hematoxylin to color the nuclei blue-purple. The samples were subsequently stained with eosin to color the cytoplasm pink. After staining, the sections were dehydrated, cleared in xylene, and covered with a glass coverslip. The stained sections were examined under a microscope to assess LN metastasis.

### Single-cell suspension preparation.

Freshly harvested mouse LNs or spleen tissues were placed in PBS and stored at 4°C. LN or spleen samples were processed within 5 hours to ensure cell viability and optimal results for downstream applications. The LNs or spleens were placed in a culture dish containing cell staining buffer (BioLegend, 420201). The flat end of a 5 mL syringe plunger was used, and the tissue was gently ground on ice to ensure thorough dissociation into cell staining buffer. The buffer was subsequently passed through a 70 μm cell strainer (SPL Life Sciences, 93040) to remove undigested tissue and debris. The filtered suspension was centrifuged at 450*g* for 5 minutes, and the supernatant was discarded. For spleen tissue, RBC lysis was necessary. The cell pellet was subsequently resuspended in cell staining buffer. Finally, the cells were counted using a hemocytometer or automated cell counter, and the single-cell suspension was ready for FCM.

### FCM.

The appropriate amount of each primary antibody was added to the prepared single-cell suspension by following the instructions for the optimal concentration. Detailed information on the antibodies used in this FCM experiment can be found in Supplemental Table 3. The cells were incubated on ice in the dark for 30 minutes. After staining, the cells were washed by adding 2 mL of cell staining buffer and were centrifuged at 450*g* for 5 minutes. The supernatant was discarded, and the pellet was resuspended in fresh staining buffer. The samples were analyzed on a flow cytometer (Beckman, C00445). The FCM gating strategies used in this study were as follows: CD4^+^ T cells, CD45^+^CD3^+^CD4^+^; CD8^+^ T cells, CD45^+^CD3^+^CD8^+^; DCs (45), CD45^+^MHCII^+^CD11c^+^; CFSE-labeled cells, CFSE^+^ cells; LTβR expression on the surface of HEVs (39), CD45^–^CD31^+^MECA-79^+^LTβR^+^; CD4^+^ Tregs, CD45^+^CD3^+^CD4^+^CD25^+^FOXP3^+^; myeloid-derived suppressor cells (MDSCs), CD11B^+^GR1^+^; activated/exhausted T cells, CD45^+^CD3^+^CD8^+^CD69^+^PD1^+^; and T cell chemokine receptors, CD45^+^CD3^+^CD4^+^CCR7^+^ (52). FlowJo software version 10.8 was used to analyze the data.

### Lymphocyte homing assay.

Briefly, according to Girard et al. (53), CFSE-labeled (BioLegend, 423801) lymphocytes were introduced into mice via the tail vein, and the LNs were removed 1–2 hours later and subjected to FCM assays for CFSE^+^ cell counts.

### ELISA.

Briefly, after the blood or cell supernatants were collected, VEGFA (MEIMIAN, MM-44452M1), VEGFC (MEIMIAN, M/M-0104M1), and VEGFD (MEIMIAN, MM-0106M1) levels were measured using specific ELISA kits. Standards and samples were added in duplicate to the wells of the ELISA plate, followed by the addition of the detection antibody. After the incubation and washing steps, a substrate solution was added for color development. The absorbance was read at 450 nm using a microplate reader, and protein concentrations were calculated based on the standard curve.

### Bioinformatic analysis.

The transcriptomes of the TDLNs and NLNs were assessed using the Illumina HiSeq 2500 platform (Illumina). The differential expression analysis was conducted with the “limma” package, identifying differentially expressed genes (DEGs) based on *P* < 0.05 and a |logFc| > 0.585. The KEGG and GO enrichment analyses were performed using the “clusterProfiler” package, with a *q* value threshold set at 0.05. According to the order of gene number, the KEGG enrichment analysis shows the top 10 terms, and the GO enrichment analysis shows the top 5 terms.

### Statistics.

The data were analyzed using GraphPad Prism (version 9.1) and R 4.4.1 software. The results are presented as the mean ± SDs. For animal and cell-based experiments, the statistical significance of differences between groups was determined using unpaired 2-tailed *t* tests for 2-group comparisons or 1-way ANOVA followed by appropriate post hoc tests for multiple comparisons. *P* < 0.05 were considered statistically significant. All experiments were performed with at least 3 independent replicates.

### Study approval.

The LN specimens used in this study were obtained with patient consent and approved by the Ethics Committee of the Second Affiliated Hospital of Nanchang University (2022 Medical Research Ethics Review No. 39). All animal procedures were conducted in accordance with the Chinese guidelines for the ethical review of laboratory animal welfare and were approved by the Animal Ethics Committee of Nanchang University (NCULAE-20221031046).

### Data availability.

Values for all data points in graphs are reported in the Supporting Data Values file. RNA-Seq data have been deposited in the Sequence Read Archive of the National Center for Biotechnology Information (https://www.ncbi.nlm.nih.gov/sra/) under BioProject number PRJNA1272038.

## Author contributions

WY contributed conceptualization, data curation, formal analysis, investigation, and methodology. JW contributed software, writing of the original draft, review, and editing. XS contributed review and editing. HX contributed data curation and investigation. SC contributed data curation, investigation, methodology, and software. JX contributed data curation. XZ contributed investigation. XY contributed data curation, investigation, methodology, validation, review, and editing.

## Supplementary Material

Supplemental data

Unedited blot and gel images

Supporting data values

## Version 1 07/22/2025

Electronic publication
