# Supplementary material for: VEGFD/VEGFR2 axis induces the dedifferentiation of high endothelial venules and impairs lymphocyte homing
Source: JCI Insight. 2025 Jul 22;10(14):e191041. doi: 10.1172/jci.insight.191041 (PMC12288975; doi:10.1172/jci.insight.191041)
Supplement: Supplemental data [file jciinsight-10-191041-s271.pdf]

Figure S1

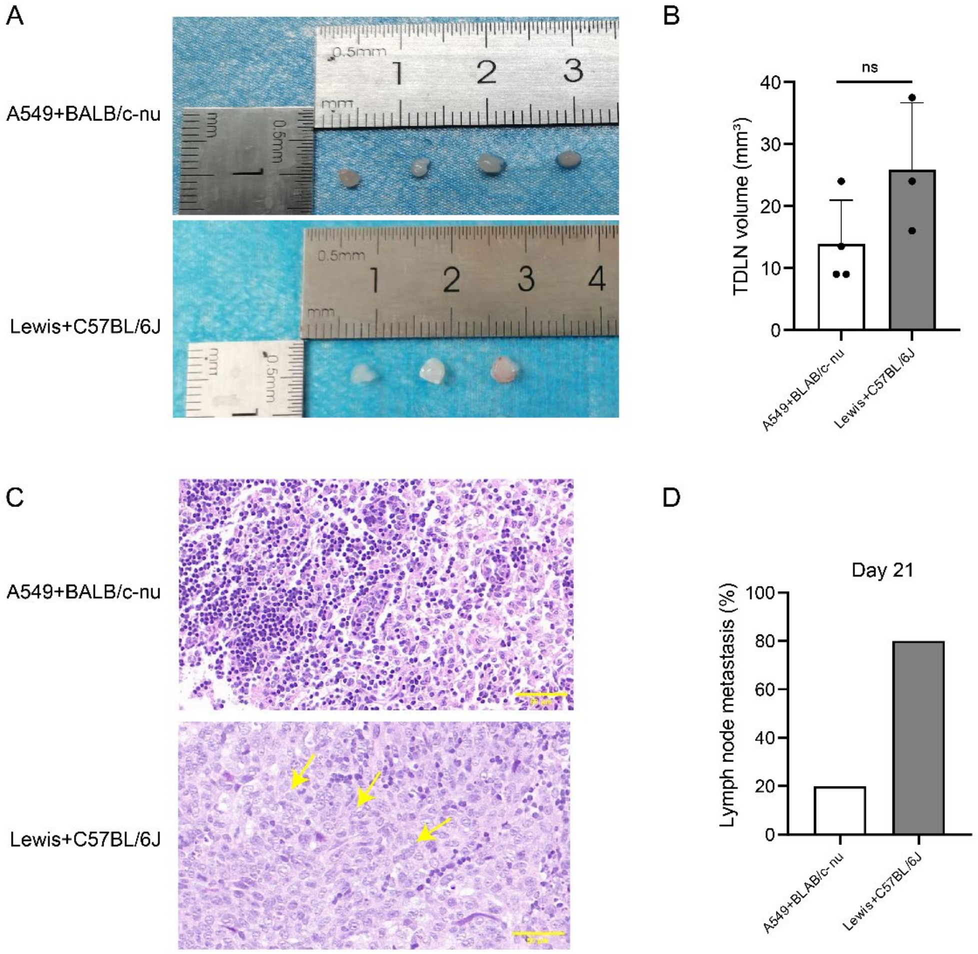

Figure S1. Construction of the TDLN model.

(A) Tumor draining lymph node (TDLN) of C57BL/6J (n=3) and BLAB/c-nu (n=4). (B) TDLN volume in C57BL/6J(n=3) and BALB/c nude(n=4) mice. (C) Hematoxylin and eosin staining of TDLN in C57BL/6J and BLAB/c-nu. (D) TDLN metastasis rate in C57BL/6J (n=10) and BALB/c nude (n=5) mice. Data are shown as mean ± SD, (B) data are percentage. (B) P values were calculated by unpaired two-tailed Student's t-test. ns: no significant.

Figure S2

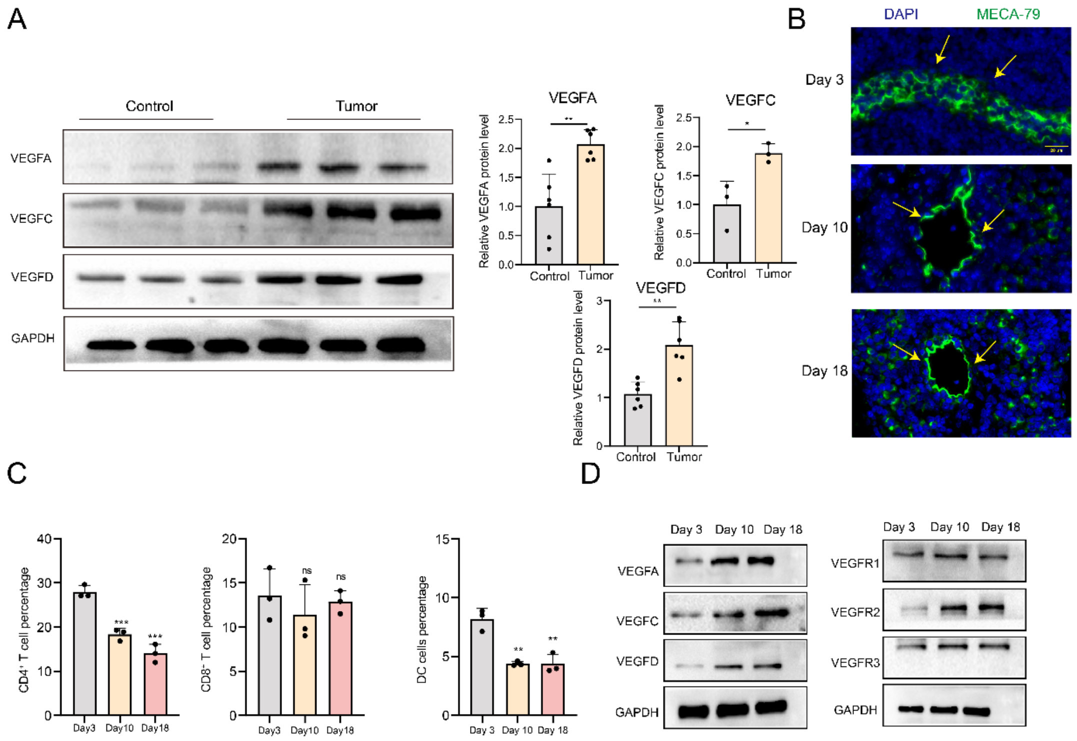

Figure S2. Changes in the tumor draining lymph node microenvironment with tumor progression.

(A) Differences in the expression of VEGFA (n=6), VEGFC (n=3) and VEGFD (n=6) in lung cancer and normal tissues were detected by western blotting. (B) Morphological changes of high endothelial venule on days 3, 10 and 18 after tumor formation. (C) Flow cytometry for percentage of $CD4^+$, $CD8^+$ T cells and dendritic cells on days 3, 10 and 18 of tumor formation (n=3). (D) Western blotting assay of VEGF-related proteins on days 3, 10, and 18 of tumor formation (n=3). (A, B) Data are shown as mean ± SD. (A) P values were measured by unpaired, two-tailed Student's t-test with or without Welch's correction analysis. (B) P values were measured by one-way ANOVA with Tukey's comparison test. *p < 0.05, **p < 0.01, ***p < 0.001, ns: no significant.

Figure S3

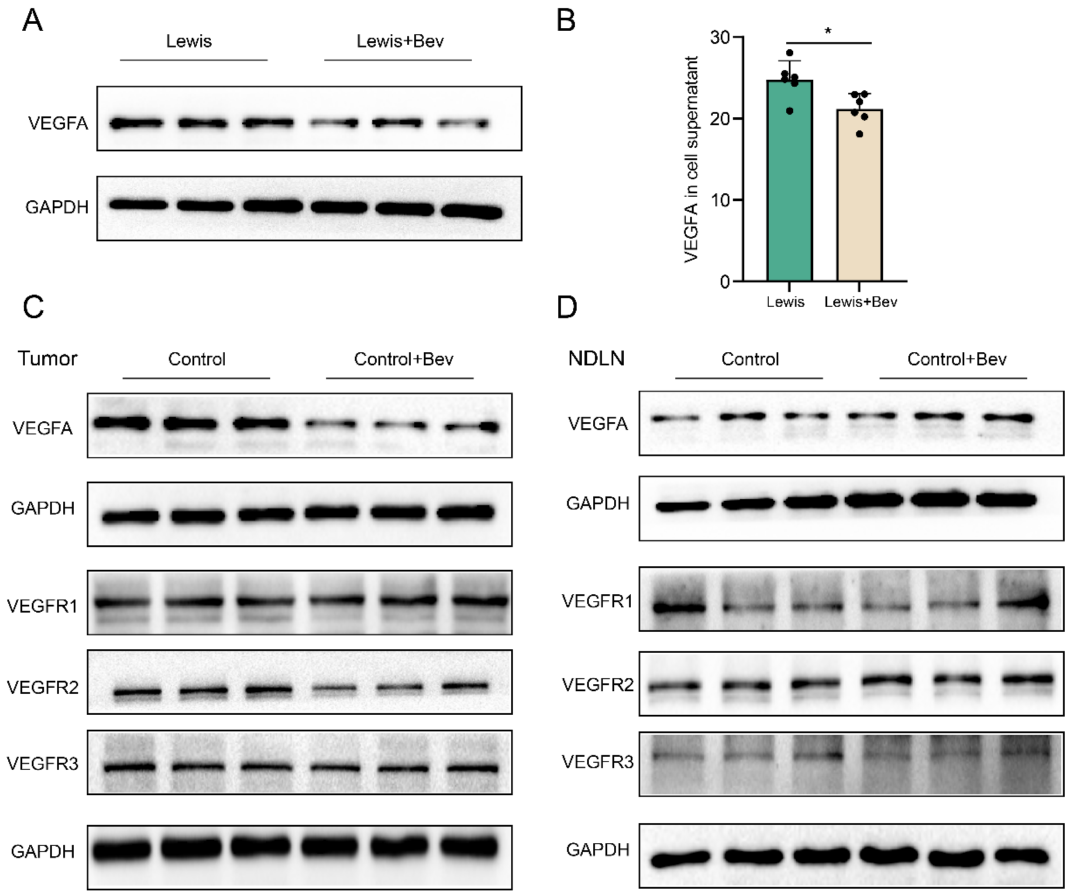

Figure S3. Bevacizumab inhibits tumor secretion of VEGFA.

(A) Western blotting (WB) assay of VEGFA in Lewis and Lewis+bevacizumab (n=3). (B) Elisa assay of VEGFA expression in cell supernatants in each group (n=6). (C) WB assay of VEGFA and its related receptor expression in tumor tissues between the control and control+bevacizumab groups (n=3). (D) WB assay of VEGFA and its related receptor expression in non-draining lymph node between the bevacizumab and control group (n=3). (B) Data are shown as mean ± SD, P values were measured by unpaired, two-tailed Student's t-test with or without Welch's correction analysis. *$p < 0.05$.

Figure S4

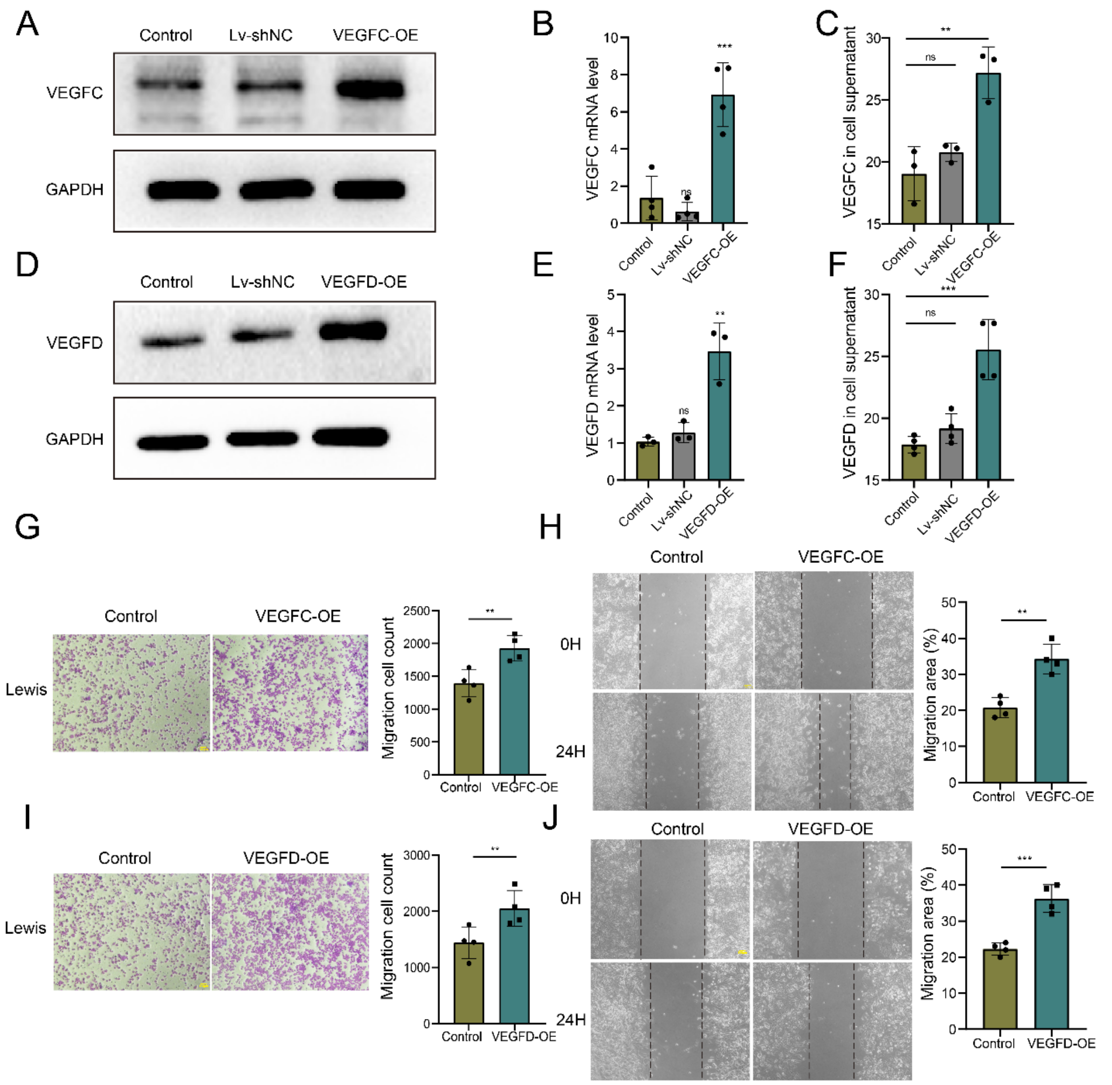

Figure S4. VEGFC and VEGFD promote lung cancer cell migration.

(A) Western blotting (WB) assay of VEGFC (n=3). (B) Quantitative reverse transcription polymerase chain reaction (qRT-PCR) assay of VEGFC (n=4). (C) Elisa assay of VEGFC expression in cell supernatant (n=3). (D) WB assay of VEGFD expression (n=3). (E) qRT-PCR assay of VEGFD (n=3). (F) Elisa assay of VEGFD expression in cell supernatant (n=4). (G) Transwell assay in the control and VEGFC overexpressing (VEGFC-OE) group (n=4). (H) Scratch assay in control and VEGFC-OE group (n=4). (I) Transwell assay in the control and VEGFD overexpressing (VEGFD-OE) group (n=4). (J) Scratch assay in control and VEGFD-OE group (n=4). (B-J) Data are shown as mean ± SD, P values were measured by unpaired, two-tailed Student's t-test with or without Welch's correction analysis. $**p < 0.01$, $***p < 0.001$, ns: no significant.

Figure S5

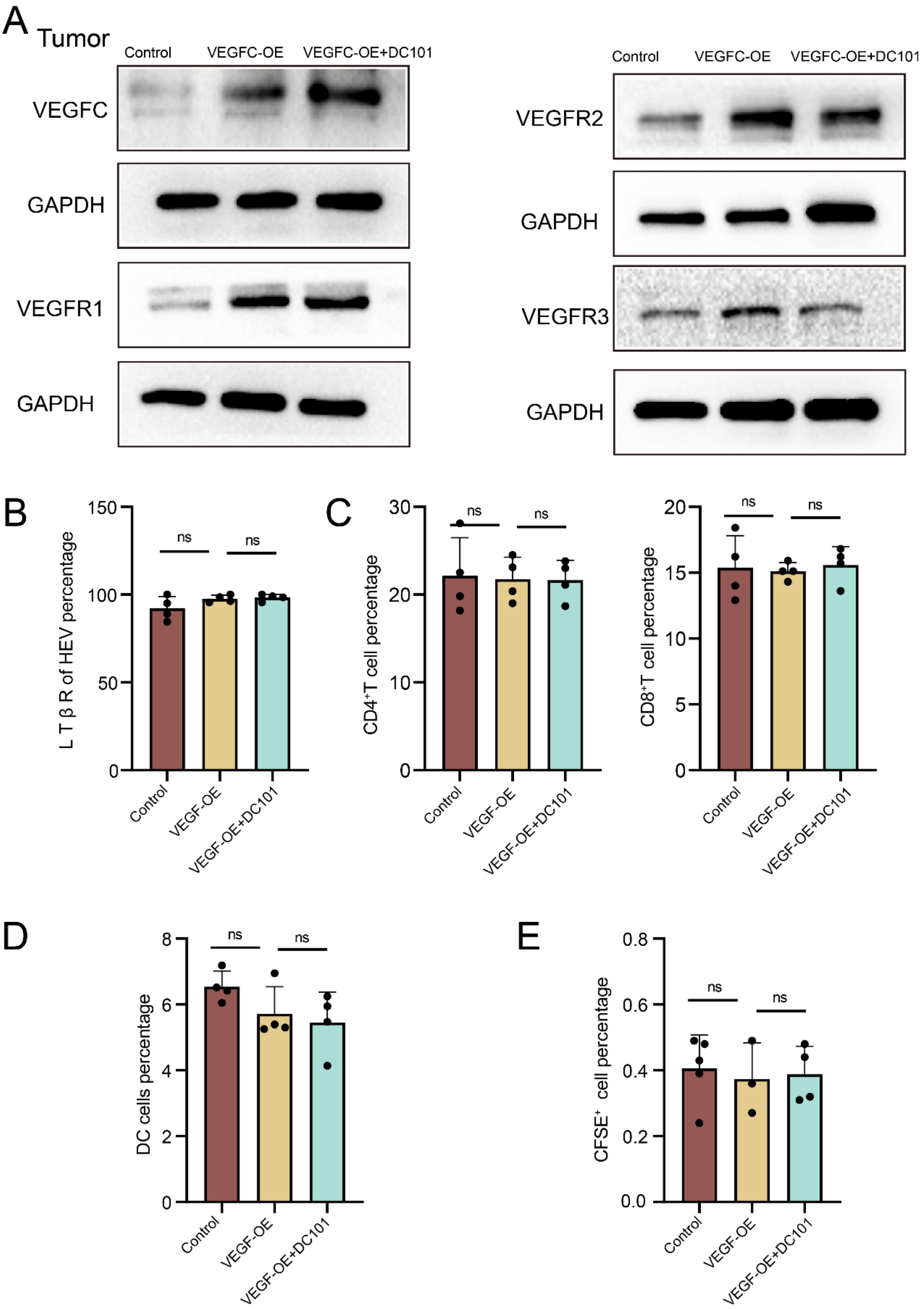

Figure S5. VEGFC-induced microenvironmental alterations.

(A) Western blotting assay of VEGFC and its associated receptor expression of tumor tissues in the control, VEGFC overexpressing (VEGFC-OE) and VEGFC-OE+DC101 groups (n=3). (B) Flow

cytometry (FCM) of LTβR expression on the surface of HEV in NDLN in each group (n=4). (C-D) FCM of $CD4^+$, $CD8^+$T cells and dendritic cells in non-draining lymph node (NDLN) between control, VEGFC-OE and VEGFC-OE+DC101 groups (n=4). (E) FCM of $CFSE^+$ cells in NDLN in each group (n=4). (B-E) Data are shown as mean ± SD, P values were measured by one-way ANOVA with Dunnet's comparison test. ns: no significant.

Figure S6

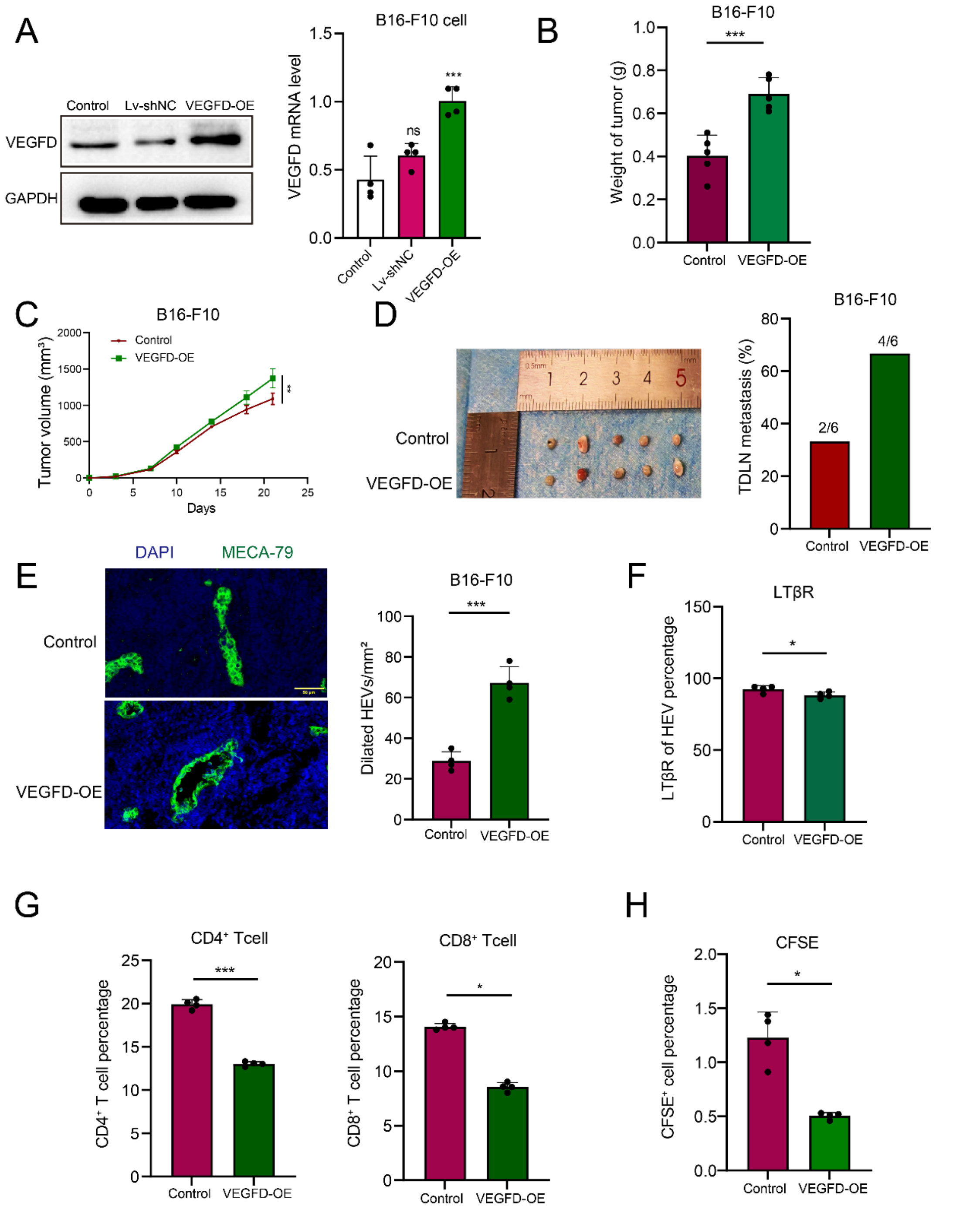

Figure S6. VEGFD overexpression promotes HEV de-differentiation in a melanoma model. (A) Western blotting and quantitative reverse transcription polymerase chain reaction (qRT-PCR) were performed to detect VEGFD expression in B16-F10 cell. (B) Tumor weight in the control and VEGFD-OE groups (n=5). (C) Tumor growth curves in the control and VEGFD-OE groups (n=5) (D) Tumor draining lymph node (TDLN) metastasis rate in the control and VEGFD-OE groups (n=6). (E) Immunohistofluorescence staining of MECA-79 (green) in TDLN between two groups (n=4). (F) Flow cytometry (FCM) of LTβR expression on the surface of HEV between the control and VEGFD-OE groups (n=4). (G) The percentage of $CD4^+$ and $CD8^+$T cells in control and

VEGFD-OE groups by FCM (n=4). (I) The percentage of $CFSE^{+}$ cells in in control and VEGFD-OE groups by FCM (n=4). In this figure, data are shown as means ± SD. (A) P value measured by one-way ANOVA with Dunnet's comparison test. (B-H) P values were measured by unpaired, two-tailed Student's t-test with or without Welch's correction analysis. *$p < 0.05$, **$p < 0.01$, ***$p < 0.001$, ns: no significant.

Table S1 The basic characteristics of lung cancer patients.

	Paitent 1	Paitent 2	Paitent 3	Paitent 4	Paitent 5
Gender	Male	Male	Female	Male	Male
Age	58	61	56	67	53
Smoking	Yes	Yes	No	Yes	Yes
Pathological	NSCLC	NSCLC	NSCLC	NSCLC	NSCLC
TNM stage	T2N1M0	T1N1M0	T3N1M0	T1N2M0	T2N1M0
Hypertension	No	No	No	No	No
Diabetes	No	No	No	No	No
Genetic mutation	EGFR	EGFR	ALK	No	EGFR

NSCLC: Non-small cell lung cancer.

Table S2 The primer sequences.

Gene	Forward Primer	Reverse Primer
GAPDH	AGGTCGGTGTGAACGGATTTG	GGGGTCGTTGATGGCAACA
Chst4	GGGTTCCCAGGTCATCGTTG	CCGAAAAGCTGTCCCACAAAA
Macadm1	CCTGGCCCTAGTACCCTACC	CCGTACAGAGAGGATACTGCTG
Fut7	AGCTGGAGGAGCAACATTCAT	GGATGGTGAGTGTGGACTGAG
Glycam1	GTCCTGCTATTTGTCAGTCTTGC	CCTGGGCCTCTTGATTCTCTG
VEGFC	GAGGTCAAGGCTTTTGAAGGC	CTGTCCTGGTATTGAGGGTGG
VEGFD	CTCCACCAGATTTGCGGCAACT	ACTGGCGACTTCTACGCATGTC

Table S3 The information regarding the antibodies used in this study.

Antibodies	Source	Catalog Number
**Western blotting/ Immunohistochemical / Immunohistofluorescence**		
GAPDH	Proteintech	60004-1-Ig
VEGFA	Proteintech	19003-1-AP
VEGFC	Proteintech	22601-1-AP
VEGFD	Proteintech	26915-1-AP
VEGFR1	Proteintech	13687-1-AP
VEGFR2	Proteintech	26415-1-AP
VEGFR3	Abcam	ab300403
LTβR	Proteintech	20331-1-AP
CD4	Abcam	ab288724
MECA-79	Nouvs	NBP2-78792
CCL21	R&D Systems	AF457-SP
CCL19	Thermo Fisher	PA5-109488
CD31	Cell Signaling Technology	77699S
LYVE-1	Abcam	ab218535
CD11c	Cell Signaling Technology	45581T
CD3	Santa Cruz	sc-20047
CK	Santa Cruz	sc-81714
**Flow cytometry**		
MECA-79	Santa Cruz	sc-19602
CD45	Biolegend	157607
CD3	Biolegend	100219
CD4	Biolegend	1004111
CD8	Biolegend	100707
CD11c	Biolegend	117309
MHCII	Biolegend	107607
CD11B	Biolegend	101211
LTβR	Biolegend	134409
GR1	Biolegend	108405
CD25	Biolegend	102043
FOXP3	Biolegend	126404
CCR7	Biolegend	120123
CD69	Biolegend	104513
PD1	Biolegend	109109
