# Supplementary material for: VEGFD/VEGFR2 axis induces the dedifferentiation of high endothelial venules and impairs lymphocyte homing
Source: JCI Insight. 2025 Jul 22;10(14):e191041. doi: 10.1172/jci.insight.191041 (PMC12288975; doi:10.1172/jci.insight.191041)

Unedited blot and gel images

Figure 2G

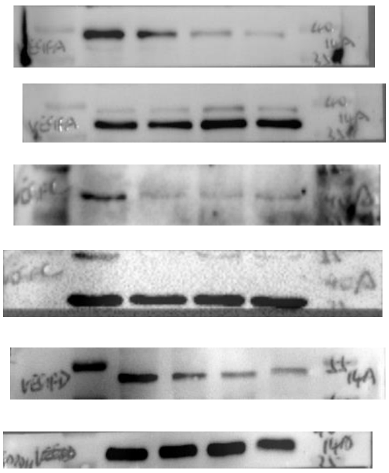

Figure 2H

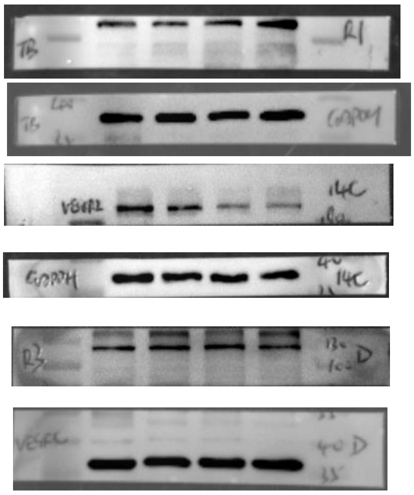

Figure 3E

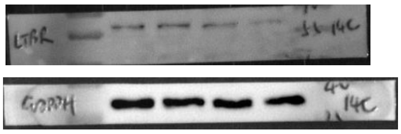

Figure 4F

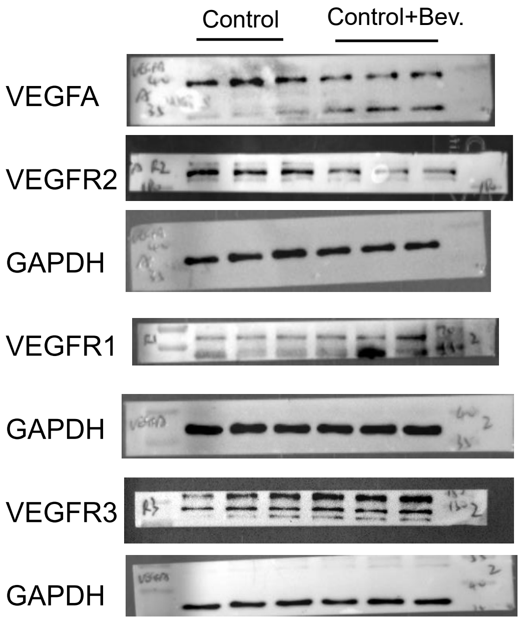
Control
Control+Bev.
VEGFA
VEGFR2
GAPDH
VEGFR1
GAPDH
VEGFR3
GAPDH

Figure 5E

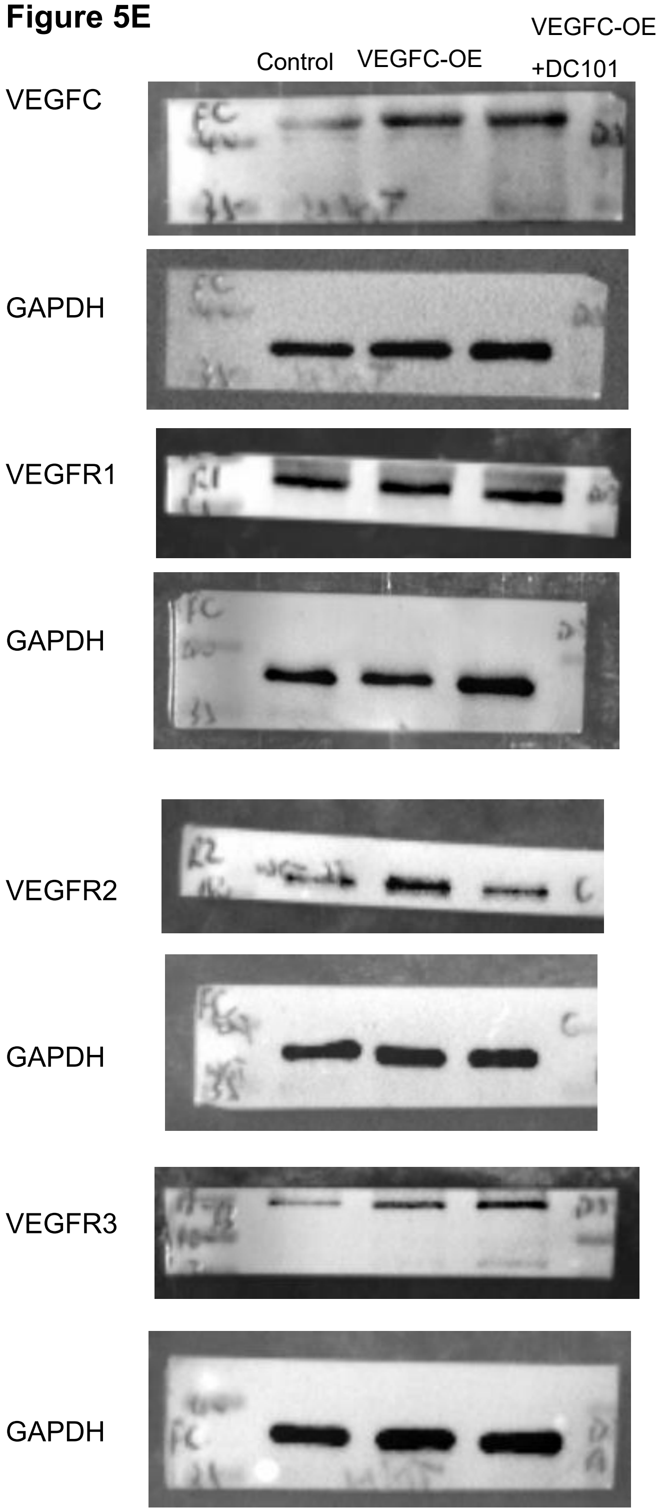
Control
VEGFC-OE
VEGFC-OE +DC101
VEGFC
GAPDH
VEGFR1
GAPDH
VEGFR2
GAPDH
VEGFR3
GAPDH

Figure 6F

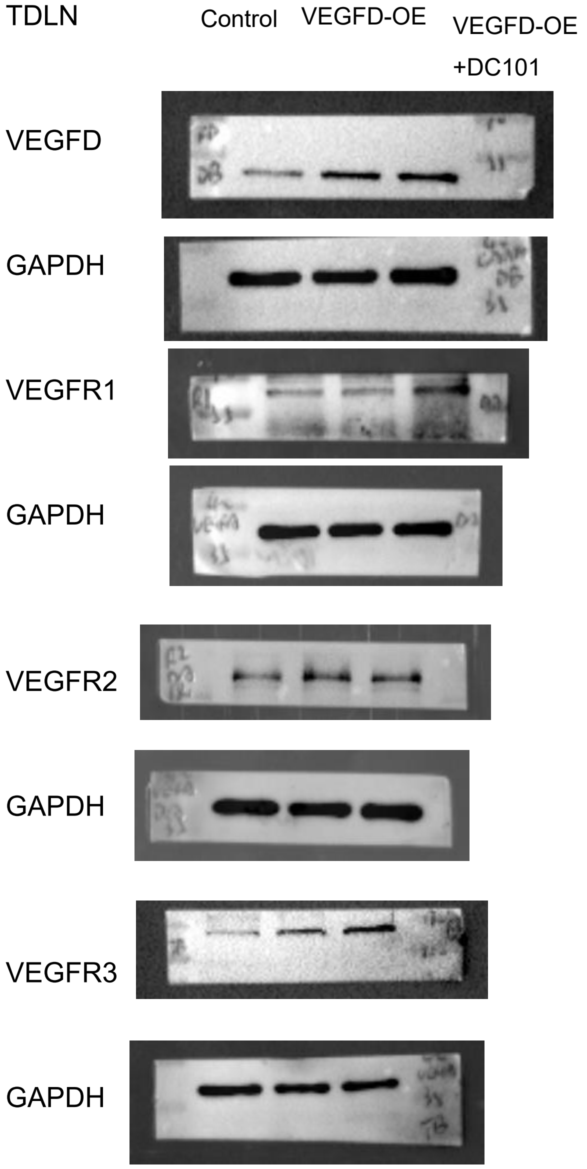

Figure 6G

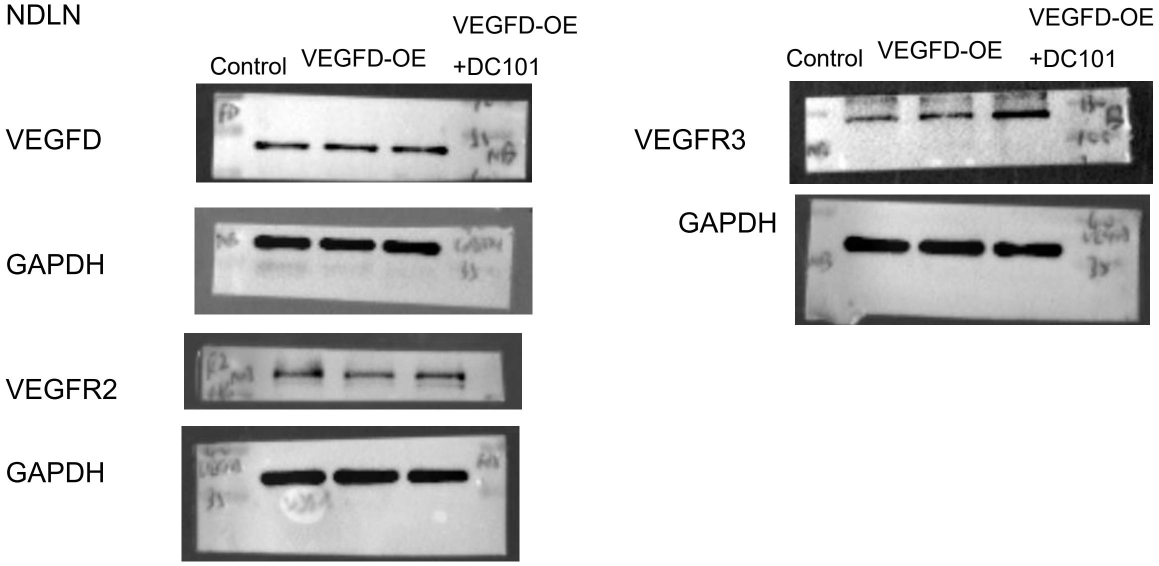

Figure 7A

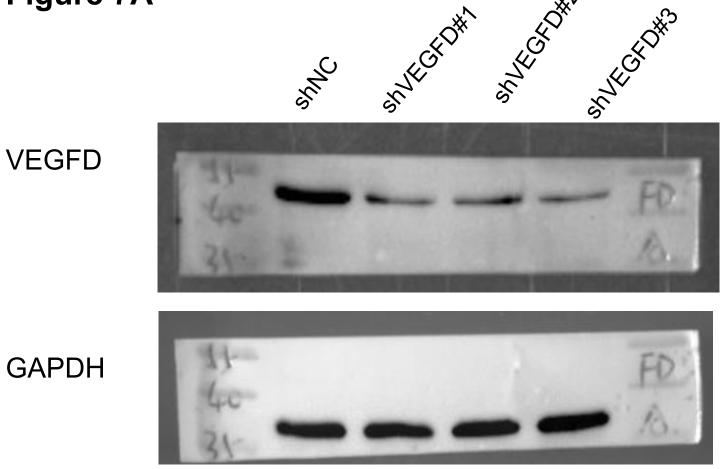

Figure S2A

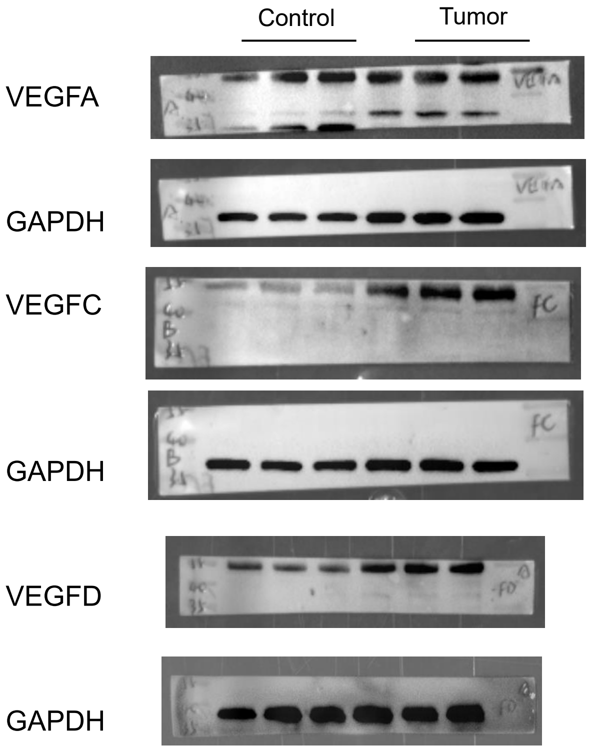

Figure S2D

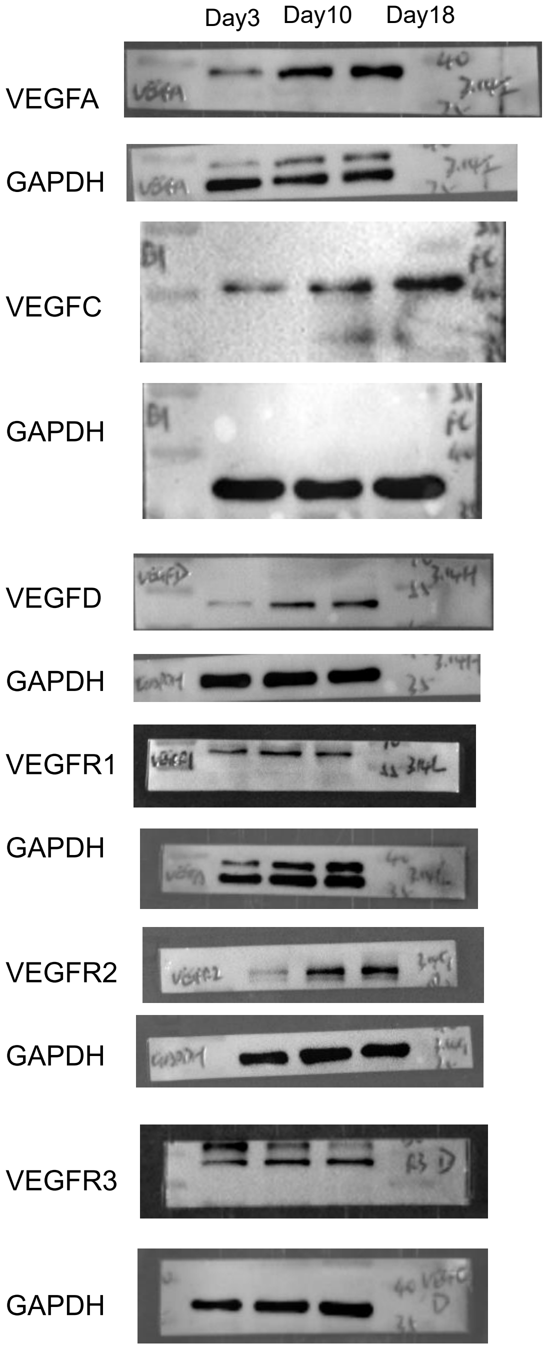
Day3
Day10
Day18
VEGFA
GAPDH
VEGFC
GAPDH
VEGFD
GAPDH
VEGFR1
GAPDH
VEGFR2
GAPDH
VEGFR3
GAPDH

Figure S3A

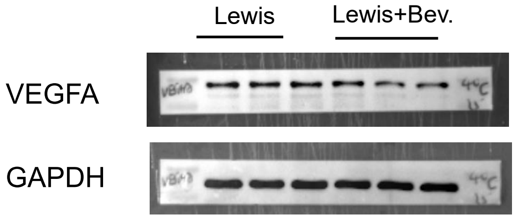

Figure S3C

Tumor

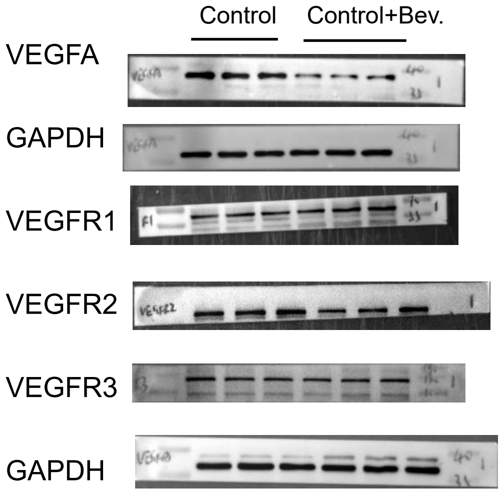

Figure S3D

NDLN

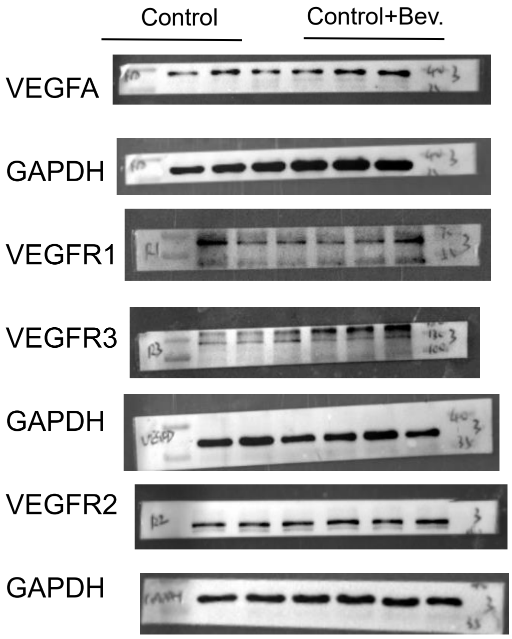

Figure S4A

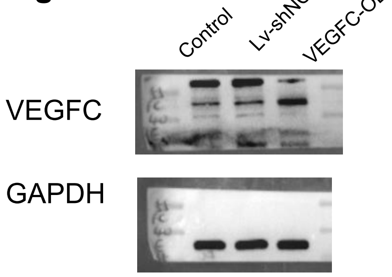

Figure S4D

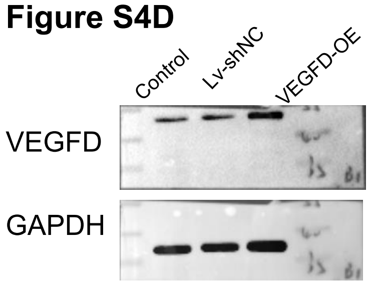

Figure S5A

Tumor

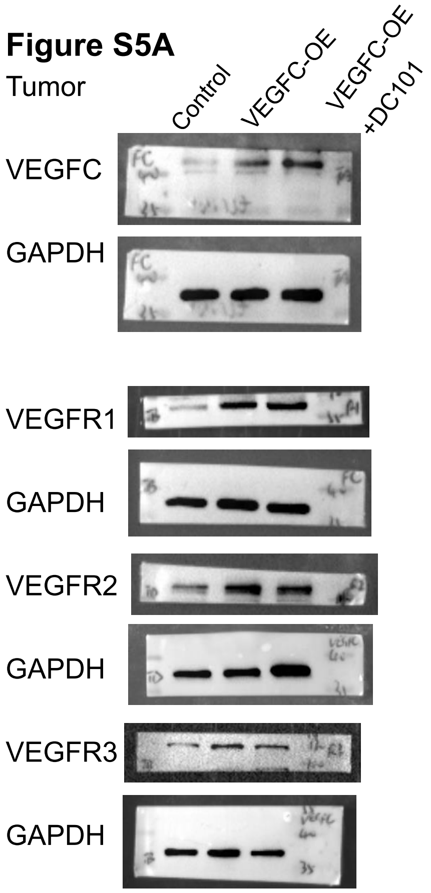

Figure S6A

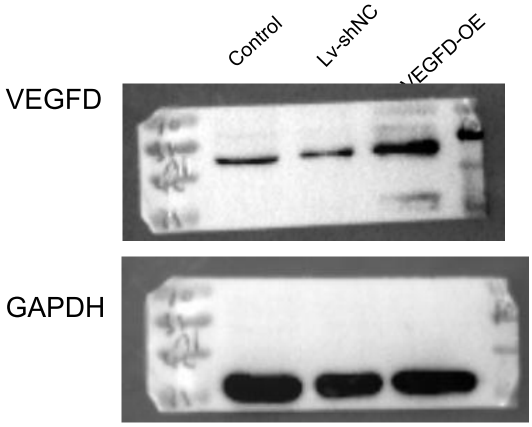

Supplement: Unedited blot and gel images [file jciinsight-10-191041-s272.pdf]
